# Justifying an Invasion: When Is Disinformation Successful?

**DOI:** 10.1080/10584609.2024.2352483

**Published:** 2024-05-22

**Authors:** Jan Zilinsky, Yannis Theocharis, Franziska Pradel, Marina Tulin, Claes de Vreese, Toril Aalberg, Ana Sofía Cardenal, Nicoleta Corbu, Frank Esser, Luisa Gehle, Denis Halagiera, Michael Hameleers, David Nicolas Hopmann, Karolina Koc-Michalska, Jörg Matthes, Christian Schemer, Václav Štětka, Jesper Strömbäck, Ludovic Terren, Sergio Splendore, James Stanyer, Agnieszka Stępińska, Peter Van Aelst, Alon Zoizner

**Affiliations:** a Department of Governance, Technical University of Munich, Munich, Germany; b Faculty of Social and Behavioral Sciences, University of Amsterdam, Amsterdam, Netherlands; c Political Communication, Universiteit van Amsterdam, Amsterdam, Netherlands; d Department of Political Science, Universiteit Antwerpen, Antwerpen, Belgium; e Political Science and Public Management, Syddansk Universitet, Odense, Denmark; f Faculty of Communication and Public Relations, Scoala Nationala de Studii Politice si Administrative, Bucuresti, Romania; g Law and Political Science, Universitat Oberta de Catalunya, Barcelona, Spain; h Journalism Media and Communication, University of Gothenburg, Goteborg, Sweden; i Faculty of Social Sciences, University of Haifa, Haifa, Israel; j Social Communication, Uniwersytet im Adama Mickiewicza w Poznaniu, Poznan, Poland; k Political Communication, Johannes Gutenberg University Mainz, Mainz, Germany; l Political Communication & Journalism, Universiteit van Amsterdam, Amsterdam, Netherlands; m International & Comparative Media Research, Universität Zurich, Zurich, Switzerland; n Communication, Universität Wien, Vienna, Austria; o Communication and Media, Loughborough University, London, UK; p Sociology and Political Science, Norges teknisk-naturvitenskapelige universitet, Trondheim, Norway; q Communication, culture & languages department, Audencia Business School, Nantes, France; r Social and Political Sciences, Universita degli Studi di Milano, Milano, Italy

**Keywords:** Conspiracy thinking, social media, public opinion, Russia, Ukraine, conflict

## Abstract

Conventional wisdom suggests that social media, especially when used by authoritarian powers with nefarious aims, leaves citizens of democratic countries vulnerable to psychological influence campaigns. But such concerns overlook predispositions among recipients of false claims to reject (or to endorse) conspiratorial narratives. Analyzing responses from a survey fielded in 19 countries, we find that it is a preexisting conspiracy outlook at the individual level, more so than media diets, which consistently predicts rating Russia’s pretenses for the invasion as more accurate. In all countries, individuals who view the world in general with a conspiratorial mindset are more likely to believe war-related disinformation. Receiving news via YouTube, Facebook, or TikTok is also associated with greater belief in Russian narratives justifying the invasion in several countries, but these relationships are weaker compared to those observed for conspiracy thinking. Without downplaying a potential positive role for media interventions, the findings highlight the importance of a deeper understanding of the underlying causes of conspiratorial thinking.

”The Kremlin is intentionally spreading outright lies that the United States and Ukraine are conducting chemical and biological weapons activities in Ukraine. We have also seen PRC officials echo these conspiracy theories. This Russian disinformation is total nonsense and not the first time Russia has invented such false claims against another country. Also, these claims have been debunked conclusively and repeatedly over many years.”

- U.S. Department of State, March 9, 2022

Russia’s disinformation machinery, widely perceived to have attempted to influence election outcomes in Western democracies (U.S. Senate Select Committee on Intelligence, 2017),^1^ has found a fertile environment on social media. Social media, used today by large majorities in all European societies and beyond, could be expected to be an important ally of Russia’s efforts to win the informational war for several reasons. They are venues for social interaction and information consumption without gatekeepers; they have a capacity for creating informational cascades and rapid scaling; social media algorithms are prone to prioritizing emotional and eye-catching content; and social media companies’ investments to stop the proliferation of disinformation in a timely manner are believed to be insufficient. All these factors make social media users potentially vulnerable to orchestrated disinformation campaigns, conceivably influencing international support for Ukraine, inasmuch as direct aid or sanctions may hinge on public opinion in countries which currently provide it with some form of assistance. But has Russia’s international disinformation campaign succeeded at convincing majorities outside of Russia about the truthfulness of narratives justifying its invasion of Ukraine? Existing research in the American context finds little support for the idea that social media users consume mis- or disinformation in mass, with both production and consumption of false information on social media platforms being heavily concentrated amongst a small group of older, conservative users (Eady et al., 2023; Guess et al., 2018; Guess et al., 2019). What are, then, the main determinants of endorsing misinformation about the war?

In this paper, we show that while Russia wages a sophisticated and multi-platform informational war,^2^ its efforts remain largely unsuccessful at implanting pro-Russian beliefs among the broader public in European societies. We build on previous research pointing at technological, media, socioeconomic and psychological determinants for endorsing disinformation. Specifically, we explore the role of social media platforms, mainstream media consumption, socioeconomic resources and demographics, and seeing the world in conspiratorial terms as predictors of endorsing disinformation. We theorize that, while endorsement of grossly inaccurate and debunked statements propagated by the Kremlin (such as that Ukraine’s government is controlled by Neo-Nazis and that the U.S. is funding biological weapons research in Ukraine) are likely associated with (social) media use and other individual attributes and demographics, they are most likely to resonate among people who have a preexisting conspiracy-oriented mindset.

We fielded a 19-country survey two months into the Russia-Ukraine war aiming to gauge the public’s endorsement of debunked narratives put out by Russia and actively circulated on social media. Our study has a unique geographic spread, spanning numerous European countries – including many understudied cases such as Greece, Serbia, Hungary, Czechia, and Poland – as well as Brazil and the United States. Building on existing literature proposing different theoretical ideas as to the most critical determinants for endorsing disinformation, we use a rich battery of questions concerning media diets, individual attributes, and demographic information to test whether holding a conspiratorial view of the world is predictive of beliefs in Russia-sponsored disinformation. Our findings show that while publics in most societies under study do not endorse false narratives about the invasion, large subsets of citizens in a small group of countries exhibit a strong tendency to evaluate disinformation as truthful. Contrary to the decisive role attributed to social media for endorsing disinformation in public narratives, our analysis shows that holding a conspiratorial view of the world is a more important contributor than social media use, political cynicism and a number of other individual-level attributes and behaviors. We show explicitly that one would drastically overestimate the influence of social media in the absence of data about respondents’ conspiratorial predispositions. While the available pool of anti-system thinkers may seem to contain both cynics and people with a conspiracy mindset, we also find that it is the latter group that is willing to endorse pro-Russian narratives during the early stages of the invasion.

## Social Media and Disinformation

Disinformation – defined here as “a subset of misinformation that is deliberately propagated” (A. Guess & Lyons, 2020, p. 10) – regarding Russia’s invasion of Ukraine only comes to further inflate a prevalent phenomenon on social media in relation to a variety of topics, from politics to diseases and health issues. The proliferation of disinformation, regardless of whether it is produced by an independent or a paid (foreign) agent, a bot, a dedicated news website, a conspiracy theorist or hyperpartisan media,^3^ is an unavoidable part of the contemporary information environment. To be sure, it is not a new development: since the early days of the internet, websites with varying levels of truthfulness were published (Iyengar & Massey, 2018). What *is*, however, new is the capacity of actors benefiting from the circulation of disinformation to amplify this type of content and help it reach a wider audience. Most Americans believe that made-up news causes a great deal of confusion (Pew Research Center, 2019), half of the U.S. public expresses worries about false and misleading information online (Knight Foundation, 2022), underlining that an information ecosystem structured around digital platforms may reduce the quality of the average news item encountered by users and, as a consequence, the capacity to make well-informed political decisions and evaluations.

Concerns about the role of social media in spreading disinformation are far from an American phenomenon. Fears that disinformation can be detrimental are highlighted by the fact that about half of the public in Europe, North America, Asia and Latin America worry about being able to identify the difference between what is real and fake on the internet when it comes to news (Newman et al., 2022). Theoretically, falsehoods can affect the audience directly, by affecting people’s information diets upon which they base their policy preferences and choices, or indirectly by fostering, for example, political cynicism, political polarization and out-group animosity among other negative developments. At the same time, although there is agreement that disinformation circulates in social media environments, especially in periods of heated events like elections or wars, studies on the impact of disinformation on individual-level attributes critical for democratic choices, such as political trust and knowledge, are scarce (Tucker et al., 2018, p. 15).

Causal effects of exposure to disinformation remain difficult to identify but observational evidence shows that beliefs in disinformation, like deliberately created and distributed conspiracy theories, are associated with individual factors, i.e., predispositions like conspiracy thinking (Enders et al., 2020; Uscinski et al., 2021). Individuals’ preexisting conspiratorial orientations and the extent to which (false) information is congruent with prior beliefs (Enders et al., 2022) seem to shape citizens’ beliefs about political phenomena. Contextual factors may either reduce or amplify individuals’ vulnerability to believe in false information (Ognyanova et al., 2020; Uscinski et al., 2022). Moreover, particularly during a war, local proximity to military events subject to disinformation and local propaganda can mitigate misperceptions about the event given the access to first-hand experiences – also for those more susceptible to believe in disinformation (Silverman et al., 2021).

Russia’s invasion of Ukraine in 2022, an event of critical international significance, has been accompanied by a deluge of streaming disinformation circulated both domestically in the form of state propaganda and internationally and in various forms (text, image, video, sound), taking advantage of the affordances of effectively all available popular social media platforms. In fact, Russia has long made Ukraine the epicenter of its domestic and international propaganda. Studies about disinformation campaigns waged over Ukraine show that they may not be only among the most advanced waged by Russia, but also among the most long-lasting, with efforts to manipulate publics dating back to the early 2000s (Lange-Ionatamišvili, 2015), and even with some partial success in confusing the Ukrainian public (Erlich & Garner, 2021). The timing of our surveys provides a unique research opportunity to study the endorsement of disinformation based on narratives that are particular to the invasion and which have been demonstrably developed and systematically propagated by Russia to justify it (compared to nonviolent contexts, anxieties and perceived threats arising from an ongoing war might make individuals more prone to believing in disinformation (Kelly & Benjamin, 2017)).

Whether Russia’s information operations are effective matters not only for current policies and their durability (e.g., support for sanctions) or the political fallout from economic hardships (e.g., potential electoral costs suffered by incumbent governments due to inflation). If foreign influence operations succeed at influencing/manipulating Western public opinion, they could embolden future cyber efforts and information warfare, undermining confidence in democratic regimes (Hamilton, 2019) and their information ecosystems. For these reasons, the extent of Russia’s “success at winning the information war” continues to be a matter of both scholarly and public interest and debate.^4^

We therefore ask the following research questions:

RQ1: How does the endorsement of Russia-propagated disinformation about the invasion of Ukraine vary across countries?

RQ2: To what extent do individual predispositions (such as conspiracy thinking) and behaviors (media consumption) account for the propensity to believe in Russian narratives justifying the invasion?

## Media Consumption and Other Correlates of Endorsing Misinformation

We examine technological, media, socioeconomic and psychological determinants for endorsing disinformation. Social media has been repeatedly described as a technology whose features make it most prone to amplifying misinformation more than information (Allen, 2022). There is a variety of reasons why social media technology might do that. An important one is that certain platforms might have certain features or affordances that help disinformation proliferate more easily than in other media (such as encouraging particular followership structures and content moderation practices). It is, however, essential to note that different social media platforms vary greatly, not only in the type of affordances embedded in their architecture, but also in the degree to which these affordances exist within them, as these factors might imply different attitudinal and behavioral outcomes on users. Another reason is the platforms’ own business models, which might amplify harmful content like disinformation. Internal Facebook research, for example, offered evidence that content on the platform polarized users and research about features that increased revenue with harmful effects was stopped (Horwitz & Seetharaman, 2020). Based on this, platform affordances and business practices make it theoretically plausible that the crafting of elaborate campaigns for the diffusion of disinformation on social media would make this technology a key enabler of beliefs in unverifiable claims and narratives.

But this idea is only partially substantiated by empirical evidence. Based on existing research, social media can indeed facilitate diffusion of mis- and disinformation by allowing people to easily connect with friends, acquaintances and, depending on the platform, unknown and potentially significant others like celebrities or politicians who have wide audiences and trigger lively commentary (for a detailed discussion see Jungherr et al. (2020)). Platforms also allow users to become their own content creators, give them the opportunity to become accidentally exposed to diverse information (with varying degrees of credibility), self-select into the type of news and other types of content that fits their ideological leanings and their worldview more generally, and interact with like-minded others (Barberá et al., 2015; Eady et al., 2019; Kim et al., 2021). And while empirical evidence shows that self-selection is counterbalanced by the fact that social media offer diverse media diets – thus preventing the vast majority of users from becoming enclosed in “echo chambers” (Fletcher & Kleis Nielsen, 2018; Fletcher et al., 2021), existing work also demonstrates an association between social media use, conspiracy theories and misinformation (Enders, Uscinski, Seelig, et al., 2021; Jin et al., 2024), though the extent to which this relationship exists might depend on the platform, its individual affordances and the type of users using it (Theocharis et al., 2021).

At the same time, evidence showing social media use *causing* beliefs in disinformation has been hard to come by. Substantial work on media effects across many years has shown that people’s individual-level attributes, such as partisan identity, are likely capable of shielding them from possible direct effects, making them less gullible to misinformation and other false information than assumed (Stroud et al., 2017). Past work has especially stressed the role of motivated reasoning in seeking out and accepting false information (Kahan et al., 2017; Swire et al., 2017), suggesting that individual predispositions, rather than social media use, may play a critical role in interpreting current highly salient events such as Russia’s invasion of Ukraine through a particular lens that fits the individual’s outlook. Moreover, initial studies suggest that people’s opinions on the opposing sides in a military conflict can influence their willingness to believe false information (see Silverman et al., 2021). Against this background we expect that heavy use of social media platforms will be correlated with endorsement of disinformation (H1) and the effect will vary by the platform used (with video-sharing platforms carrying potentially more believable content).

A second set of determinants for endorsing misinformation concerns the consumption of traditional media. Theoretically, the objective of traditional media like television or newspapers has been to aid citizens in making informed choices by providing them with sufficient and relevant political information (Delli Carpini & Keeter, 1996). Especially public service media typically represent more trustworthy sources than social media due to their practice of careful curation of news and the presence of gatekeepers who are supposed to exercise journalism according to the core values of truth, factual accuracy and quality (Horowitz et al., 2022). In this sense, they have generally been thought of as a counterweight to the rampant proliferation of disinformation on social media. But as past work has discussed in some detail (Aalberg et al., 2010; Jungherr & Schroeder, 2021; Van Aelst et al., 2017), changes mainly in the political economy of the media over the last decades, including professionalizing imperatives, increased competitive pressures, the emergence of clickbait and an “outrage industry,” and changes in how people perceive news, have led to concerns and uncertainty about the extent to which traditional media fulfill this role nowadays (Freelon & Wells, 2020; Van Aelst et al., 2017). In fact, as some have argued (Tsfati et al., 2020), while traditional media are putting increasing care in properly reporting factual information (Glasser, 2016), repeating misinformation in order to correct it may, paradoxically, lead to further dissemination of misinformation. Overall, while the role of traditional media in the spread of mis- and disinformation is still not well understood (Tsfati et al., 2020), research does suggest that in times of crises people have a strong need for orientation and value quality journalism that is fast and accurate, though this is mainly observed for those who trust such media (Van Aelst et al., 2021). Evidence showing that people turned to trustworthy news outlets that typically uphold the core values of journalistic coverage during the recent pandemic (Altay et al., 2022) could mean that traditional media may shielded against mis- and disinformation in times of crisis. This leads us to our second hypothesis: reliance on traditional media will be negatively correlated with endorsement of disinformation (H2).

A final set of determinants that past work has found to play an important role in endorsing mis- and disinformation revolves around psychological, demographic and resource-based individual-level characteristics. Two strands of past work related to conspiracy theories (e.g., Enders, Uscinski, Klofstad, et al., 2021; Imhoff et al., 2022) and exposure effects (e.g., Guess et al., 2018; Ognyanova et al., 2020) are of particular relevance here. Research argues that holding a conspiratorial worldview of the world, that is, having a general predisposition to interpret salient events and circumstances as the product of malevolent forces, is an important predictor when it comes to endorsing disinformation.

We further theorize that, if seeing the world in conspiratorial terms consists of a belief system further composed of anti-establishment and anti-system beliefs as past scholarly work has suggested (Enders, Uscinski, Klofstad, et al., 2021; Uscinski et al., 2021), then political cynicism – holding politicians and the political system more broadly in disrepute – will also play an important role in shaping who endorses disinformation.^5^

Finally, past scholarly work focused on the United States reports strong age effects (Grinberg et al., 2019; Guess et al., 2019), and higher age has been a documented predictor of sharing or viewing so-called fake news.

Our dataset from 19 countries provides a rare opportunity to assess if a number of demographic characteristics play an equally important role beyond the American context in endorsing disinformation. Therefore, we hypothesize that a conspiratorial worldview and political cynicism will be positively correlated with the endorsement of disinformation (H3) and age will be positively correlated with the endorsement of disinformation.

## Data and Methods

We commissioned Kantar, a private pollster, to field surveys on our behalf in 19 countries in April-May 2022.^6^ Quota sampling was used to approximate representativeness on age, gender, and education. Details on participants’ sociodemographic characteristics per country are provided in Table SI.1 in the Supporting Information.^7^

Respondents were asked to rate the veracity of two claims which were used to justify the invasion: 1) Ukraine’s government is antisemitic and controlled by neo-Nazis; 2) the U.S. is funding biological weapons research in Ukraine. Both claims originate in popular statements (made without evidence) of Russian officials and Russian state media, and have been widely debunked by media outlets, governmental organizations, scientific groups, and international bodies, including the US Department of State.^8^ The 5-point scale ranges from 1 (very certain it’s false) to 5 (very certain it’s true). Lower values thus mean rejection of false claims while higher values correspond to greater proclivity to endorse disinformation. In most models, we average respondents’ two assessments to study the variation in a single outcome variable.

**Figure 1.**
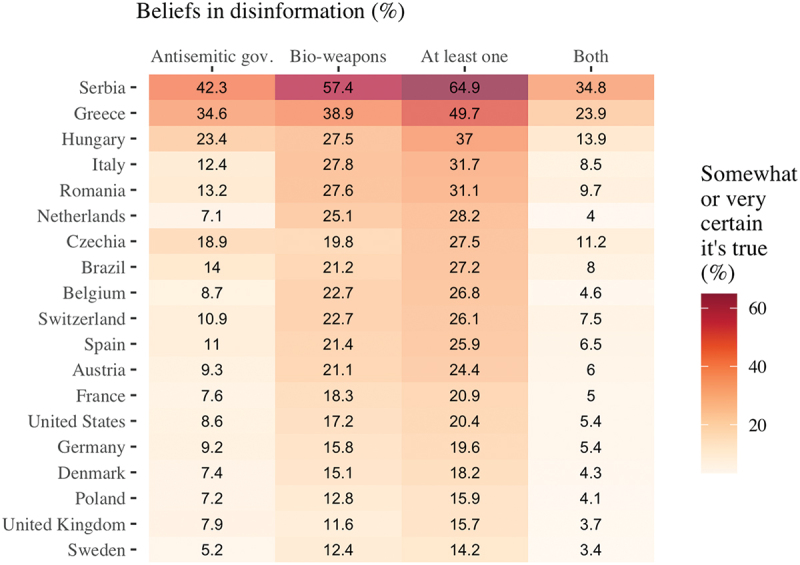
Share of respondents endorsing narratives justifying the invasion.

In all countries, at least 14% of respondents believed at least one of these two narratives (Figure 1), and in 11 countries, over 1 in 4 respondents believed in at least one of two conspiracy theories. In general, the claim about U.S. biological weapons research in Ukraine was believed by more respondents: in the pooled sample, 23% of respondents stated that the biolab theory was probably or certainly true, whereas 13.6% of respondents expressed at least some confidence in the theory that the Ukrainian government was controlled by neo-Nazis.

This information already answers RQ1 as these patterns show substantial differences *across societies*: a majority of respondents in Serbia believed at least one conspiracy theory, nearly a quarter of Greeks believed both theories, while in five countries (Poland, the U.K., Denmark, Germany, and Sweden) over 80% of respondents did not believe a single anti-Ukraine/American conspiracy theory.

Our empirical strategy is to model ratings of two (false) claims as a function of five sets of individual-level attributes. The main predictors of interest are conspiracy thinking (a scale based on a 4-item battery summarized below), political cynicism, demographic covariates (education, age and gender), as well as general media diet (i.e., respondents’ frequency of consuming news via TV, newspapers, and social media), and finally self-reported frequency of using specific platforms (Facebook, Instagram, Twitter, TikTok, or YouTube) to access news. We also estimate models where we condition on approval of the invasion to guard against the possibility that a general pro-Russia stance could confound the relationship we observe between beliefs in Russian narratives and our main predictors (i.e. conspiracy thinking, and heavy use of social media).

## Conspiracy Thinking

**Figure 2.**
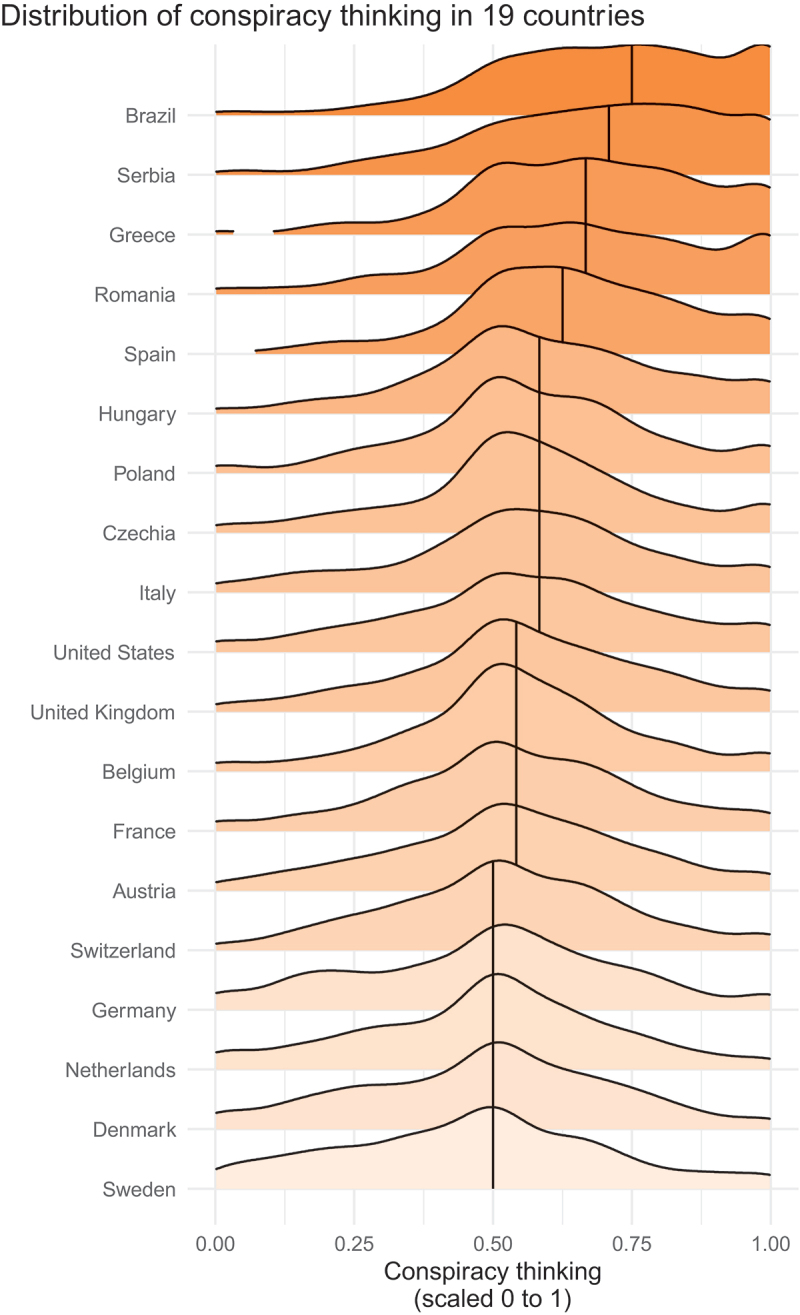
Conspiracy thinking across the sampled countries (the densities show distributions of the additive index, rescaled to range from 0 to 1; the vertical lines denote the median value for each country). The wording of four survey instruments used for these calculations, and distributions of item-by-item responses by country are shown separately in the Supporting Information (Figure SI.1).

One of our key independent variables is conspiracy thinking (Uscinski & Parent, 2014). For each country, we display density plots of the conspiracy thinking scale in Figure 2 (Cronbach’s α = 0.83), ranked by the median score (we rescale the overall score to range from 0 to 1 to facilitate interpretation of regression coefficients later). The greatest prevalence of conspiracy thinking is observed in Brazil, Serbia and Greece; Sweden, Denmark and the Netherlands rank at the bottom.

In the pooled sample, the average respondent leans slightly toward believing in general conspiracies (*M* = 0.57 on the unit scale, and *M* = 4.45 (SD = 1.4) prior to rescaling). The median country is the United States, where the extent of conspiracy thinking is comparable to Italy or the United Kingdom. The median respondent scores 0.75 in Brazil, 0.71 in Serbia, and 0.5 in the bottom five countries (Switzerland, Germany, the Netherlands, Denmark, and Sweden). These patterns are consistent with those reported by other scholars (Drochon, 2018; Smallpage et al., 2020).

We briefly describe some of the components of the scale (for extensive validation of the scale, see Uscinski et al., 2021). The least prevalent sentiment is “much of our lives are being controlled by plots hatched in secret places” with the average respondent neither agreeing nor disagreeing (*M* = 4.0, SD = 1.82, Min = 1, Max = 7).^9^ The most prevalent sentiment is that “even though we live in a democracy, a few people will always run things anyway” and the median respondent agrees at least weakly with the statement. The average response in the pooled sample of all 19 countries is 5.1 (SD = 1.54), and there is only one country (Germany) where fewer than half of respondents (48.6%) agree with the statement. The median rating of the statement is 5, and the median country for this specific instrument is Czechia, where 66% of respondents at least weakly agreed with the statement.

## Results

First, we test whether news consumption via social media predicts belief in disinformation (H1), and whether consumers of more traditional news media are better informed (H2). The first specification in Table 1 documents that respondents who frequently receive news via Facebook, YouTube and TikTok rate disinformation as more likely to be true compared to non-users. While exposure to news via Twitter is negatively correlated with beliefs in Russian narratives (a result consistent with Theocharis et al., 2021), no effect is observed once demographic and political co-variates are included in the model as controls (column 2). The table provides robust support for the first hypothesis, but as we discuss below, the magnitude of the association between news exposure via social media and beliefs in Russian propaganda is quite sensitive to model specification.

**Table 1.** Results from pooled OLS models. Cell entries are OLS coefficients and robust standard errors are in parentheses. All continuous RHS variables are scaled to range from 0 to 1. Categorical demographic variables are included as a set of dummy variables.

	Outcome variable: Average belief in disinformation					
	(1)	(2)	(3)	(4)	(5)	(6)
Platform: Facebook	0.240**	0.189**			0.119**	0.097**
	(0.026)	(0.026)			(0.025)	(0.022)
Platform: Youtube	0.228**	0.263**			0.170**	0.076**
	(0.030)	(0.030)			(0.028)	(0.025)
Platform: TikTok	0.395**	0.341**			0.277**	0.119**
	(0.034)	(0.034)			(0.032)	(0.029)
Platform: Instagram	0.046	0.013			0.015	0.042
	(0.031)	(0.032)			(0.030)	(0.027)
Platform: Twitter	−0.058*	0.021			0.036	0.004
	(0.028)	(0.029)			(0.027)	(0.024)
News: TV	−0.306**	−0.266**			−0.238**	−0.058**
	(0.024)	(0.025)			(0.024)	(0.021)
News: Newspapers	−0.272**	−0.165**			−0.115**	−0.071**
	(0.022)	(0.023)			(0.022)	(0.019)
Conspiratorial Thinking			1.447**		1.273**	0.885**
			(0.031)		(0.031)	(0.031)
Political Cynicism				0.825**		0.088*
				(0.037)		(0.035)
Univ. graduate		−0.119**			−0.081**	−0.052**
		(0.014)			(0.014)	(0.012)
Female respondent		0.158**			0.149**	0.140**
		(0.014)			(0.013)	(0.012)
Age: 18–24		−0.073*			0.004	−0.001
		(0.030)			(0.029)	(0.026)
25–34		0.021			0.044	0.025
		(0.024)			(0.023)	(0.021)
35–44		0.024			0.031	0.000
		(0.022)			(0.021)	(0.018)
55–64		0.000			−0.014	0.005
		(0.022)			(0.020)	(0.018)
65–74		−0.047*			−0.074**	−0.008
		(0.023)			(0.022)	(0.019)
75 or older		−0.093**			−0.125**	−0.030
		(0.029)			(0.027)	(0.024)
Russian operation is legitimate						0.109**
						(0.005)
Against sanctions						0.065**
						(0.004)
Russia uses disproportionate violence						−0.047**
						(0.004)
Support aid to Ukraine						−0.095**
						(0.004)
Political Interest		−0.264**			−0.230**	−0.162**
		(0.027)			(0.026)	(0.023)
Political Orientation		0.333**			0.224**	0.052*
		(0.028)			(0.027)	(0.024)
Ideological strength		0.024			0.008	0.009
		(0.022)			(0.021)	(0.018)
Country fixed effects	✓	✓	✓	✓	✓	✓
Observations	19037	19037	19037	19037	19037	19037
R^2^	0.185	0.208	0.229	0.150	0.284	0.436
RMSE	0.91	0.90	0.89	0.93	0.86	0.76

The outcome variable ranges from 1 to 5. Significance cutoffs are: * *p* < .05, ** *p* < .01.

We also find consistent support for the second hypothesis: individuals who frequently received news through television or newspapers were significantly less likely to rate disinformation as true. Model 5, for instance, where a rich set of controls is included, suggests that heavy news consumption via television is associated with a 0.2-point lower rating of disinformation on the aforementioned 5-point scale.

Our most conservative model is reported in column 6, where we also condition on proxies for sympathies with Russia or Ukraine.^10^ Adding these extra controls decreases the magnitudes of the TV and newspapers´ coefficients, but they remain informative and statistically significant.

A model predicting beliefs as a function of media consumption, education, gender, age, political orientation, and political interest (column 2) has an *R*^2^ of 0.208, and we see in column 3 that a model containing a single independent variable – conspiracy thinking – fits the data better in terms of both *R*^2^ and the root mean squared error (RMSE). Thus, turning to the third prediction (that conspiratorial and cynical predispositions contain useful signal about beliefs in propaganda) simple models (columns 3 and 4 in Table 1) seem to be promising. But the relationships in models 3 and 4 may be overstated due to omission of potential confounders. We should therefore view models 5 and 6 as the main tests of H3.

In models which condition on a rich set of media consumption variables, as well as demographic and political characteristics, a respondent with a maximum conspiracy thinking score is predicted to rate, in expectation, the accuracy of two pieces of disinformation 0.89 to 1.27 points higher (on a 5-point scale) relative to a respondent with minimal conspiracy thinking. Moreover, a respondent with maximum political cynicism is expected to rate the accuracy of disinformation 0.09 points higher relative to a respondent with minimal political cynicism.

We thus find support for H3, and we note that conspiracism is an order of magnitude more predictive of beliefs in disinformation than political cynicism. Empirical support in favor H3 remains strong even when we account for opinions tapping into evaluations of Russian military actions themselves, or respondents’ support for aiding Ukraine.

Our final hypothesis pertains to age, and we do not find support for the expectation that older respondents are more vulnerable to disinformation, which is in contrast with what U.S. studies have shown. Perhaps surprisingly, we find that the invasion is a context where older citizens are less likely to believe state-sponsored justifications of the invasion compared to the reference group (respondents born between 1968 and 1977).

**Figure 3.**
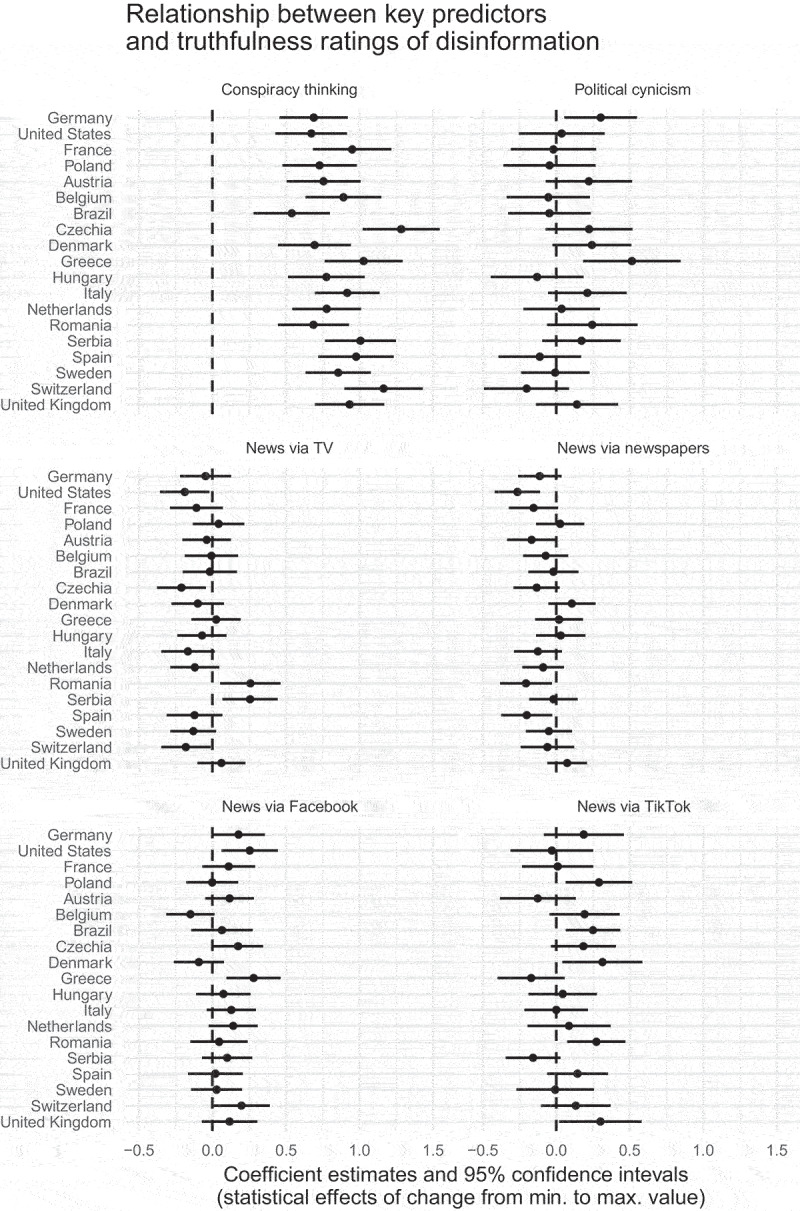
Coefficients from country-by-country regressions (using the richest specification, the pooled version of which is shown as column (6) in Table 1). Each row contains estimates from a separate model, and specific predictors of interest are pulled into six panels to facilitate cross-country comparisons. The outcome variable (average belief in disinformation narratives) ranges from 1 to 5, and all predictors are scaled to range from 0 to 1. To preserve space, some predictors (other digital platforms) and control variables (education, age, gender, political interest, and political orientation) are not displayed.

We also estimate a version of model 6 again, but instead of including country fixed effects, we use a separate model for each of the 19 countries in our sample. Plucking coefficients of interest from each of these models (Figure 3) allows us to examine differences across countries. We thus see that H1 holds in the pooled model, but it is not the case in all countries that news consumption via Facebook or TikTok is everywhere associated with greater belief in disinformation. Moreover, while the relationship between news consumption via TV and newspapers is typically negative, we also observe that there are two cases – Serbia and Romania – where respondents who watch more news via television are more likely to believe Russian invasion justifications relative to their counterparts who have low exposure to news via television in the same country.

We find the strongest and most consistent support for the expectation that a conspiratorial predisposition is associated with a greater propensity to rate disinformation as true (top left panel of Figure 3). Political cynicism is positively associated with beliefs in disinformation in countries including Germany and Greece, but the effect appears to be imprecisely estimated in many of the remaining countries (note that in the models reported in the top-right panel, conspiracy thinking is also included as one of the predictors).

Given the preceding patterns, with social media being sometimes prognostic and the conspiratorial predisposition yielding the greatest amount of signal about respondents’ beliefs, it seems fitting to ask whether social media and conspiracy thinking interact. In other words: is social media usage more likely to be associated with beliefs in disinformation among those who are prone to believe in conspiracies in general? This would be in line with U.S.-based evidence reported by Enders, Uscinski, Seelig, et al. (2021).

**Figure 4.**
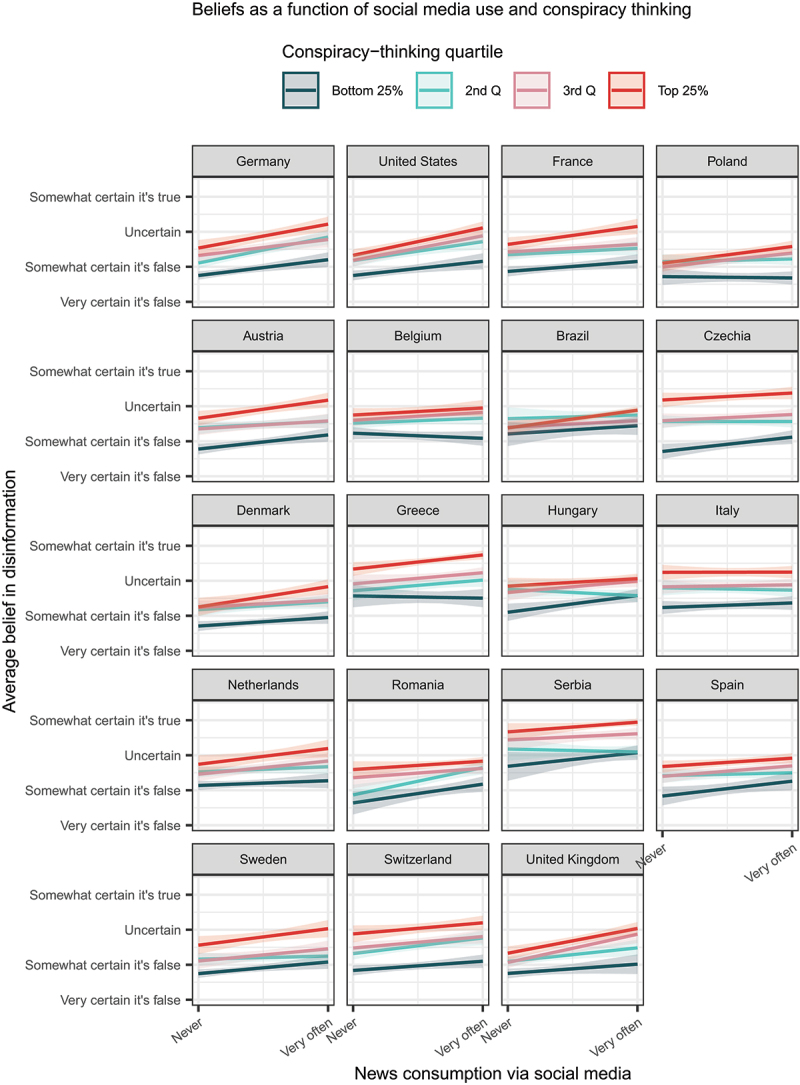
Average predicted beliefs in disinformation by news consumption via social media (interacted with the conspiracy thinking predisposition). The predictions are based on a model where conspiracy thinking is interacted with both social media consumption and country dummy variables. Controls include: political orientation (incl. its squared value), political interest, news consumption via traditional channels (TV and newspapers), education, gender, and age categories (coded in the same way as shown in Table 1).

Figure 4 shows that in several polities (Poland, the United Kingdom, and the U.S.), there is indeed an interactive effect. That said, in countries including Germany, Hungary, Austria and Spain, social media relationships are *not conditioned* by conspiracy thinking. In the pooled sample, the interaction between news consumption via social media and conspiracy thinking is significant in a model predicting beliefs in disinformation (and conditioning on demographic, political, as well as legacy media control variables), but the magnitude is relatively small; again, the key variable is conspiracy thinking and social media consumption is relatively less informative. (See also Figures SI.6, SI.7, SI.8, SI.9 where conspiracy thinking is interacted with either Facebook or TikTok use.)

## Discussion and Conclusion

Designated by *The New Yorker* as “The First TikTok War,”^11^ Russia’s invasion of Ukraine has provided a grim opportunity for further elevating social media as a battlefield for misinformation. Platforms like Facebook and TikTok have indeed been flooded with war-related misinformation and all kinds of unverified content, much of which is geared to justify an invasion routinely described by the Russian propaganda machine as a “Special Operation.” Narratives circulating on social media – as well as on Russian public television – have ranged from allegations that the Ukrainian government is run by Neo-Nazis to long de-bunked conspiracy theories about the role of Western powers in developing bioweapons in Ukraine. Richard Stengel, a former undersecretary of state for public diplomacy and public affairs, and former editor of Time Magazine, was quoted in an article published by MIT’s Sloan School of Management as having said about the Russian disinformation machine: “It’s not that they were so good, it’s that we were so susceptible. Disinformation always seeks a kind of biased audience. And people are receptive to it.”^12^ But are people indeed so susceptible? Have these efforts to mislead public opinion actually had an effect and who is this biased audience?

In this study, we had a rare opportunity to study the extent to which the European, American and Brazilian public endorsed misinformation related to Russia’s invasion of Ukraine just two months into the war. Building on existing work on the impact of conspiratorial thinking on belief in misinformation, as well as on studies finding very limited consumption of misinformation by the broader public, we theorized that not only endorsement of misinformation about Russia’s invasion is not uniform across different countries, but also that conspiratorial worldviews – not social media – are the stronger predictors of endorsing misinformation.

Our study confirms both of these expectations. While endorsements of conspiracy beliefs do not vary massively across all societies, there is a set of countries that are distinctly different than the rest when it comes to how the public evaluates the truthfulness of demonstrably false statements about the war. Moreover, and perhaps most importantly for dispelling the popular view that flags social media as the main culprits for learning about and endorsing misinformation, our findings show that holding a conspiratorial view of the world plays a larger role in endorsing misinformation about the war than using social media for news does. Indeed, while social media is generally thought of as a strong predictor of disinformation endorsement, a major finding of our study is that beliefs in Russia sponsored disinformation has, first and foremost, psychological foundations. The association between conspiratorial thinking and endorsing misinformation is such that, if conspiratorial thinking is not accounted for, the effect of social media could be overestimated by up to 150%.^13^

The study’s limitations include potential mismeasurement of disinformation beliefs due to social desirability bias,^14^ inattentiveness,^15^ or other issues,^16^ despite mitigation efforts in survey design. Another limitation is that our data lacks a temporal (panel) dimension and we are thus unable to test whether conspiracy thinking has been stable over time. While our argument is that a conspiracy mindset makes individuals more receptive to disinformation, we acknowledge that, among some individuals, repeated exposure to either misinformation or to anti-systemic content could also plausibly modify (increase) the level of the conspiracy mindset.

Lastly, the sample is not fully representative of the European Union, as it excludes several EU member states (and this omission could potentially make the EU appear more united in its support for Ukraine, given that countries like Hungary and Slovakia were not included in this study).

Given the dearth of comparative evidence in the study of misinformation, our study extends prior work on the subject by collecting evidence from the United States (the most studied case) and 18 other democracies. In contrast to studies conducted only in the U.S. suggesting that consumption and redistribution of misinformation is concentrated among older, male, and conservative users, our findings show that older respondents are less likely to endorse Russia sponsored disinformation (in some specifications the youngest respondents are among the most likely to endorse Russian narratives; see Table S.2).

Our study is consistent with previous work showing that endorsing (and potentially propagating) misinformation is concentrated among a minority of citizens, but provides a lens for identifying this minority: people with a strong conspiratorial view of the world – and not necessarily older users. This not only adds urgency to shift the focus on the determinants of disinformation consumption outside the U.S., but also toward better understanding why young people, a subgroup of the population that theoretically has the highest levels of digital literacy (but who are also avid users of TikTok and YouTube) believe some false claims at a rate at least as high as their older counterparts.

## Supplementary Material

Supplemental Material

## Notes

1. The US Senate’s Select Committee on Intelligence asserts that the Russian disinformation campaign “sought to polarize Americans on the basis of societal, ideological, and racial differences;” the operating expectation is that a more polarized society will be weaker geopolitically.

2. Deceiving narratives, as the U.S. Department of State quote at the beginning of this paper makes clear, are repeatedly propagated by public television and politicians – this is not just a social media phenomenon.

3. See Tucker et al. (2018) and Persily and Tucker (2020).

4. See, e.g., Tucker (2023), Jamieson (2020), Eady et al. (2023), Hamilton (2019), as well as the essay entitled *How to stand up to the Kremlin: Defending democracy against its enemies*, coauthored by a future U.S. president (Biden & Carpenter, 2018).

5. For the most thorough analysis to date on the correlates of conspiracy thinking, the reader is referred to Enders et al. (2023). See also Oliver and Wood (2014) and Uscinski and Parent (2014).

6. The broader research project received ethical approval by the institutional review board of the coordinating university. The questionnaire is available via OSF https://osf.io/pruda/?view_only=188fca5107ca40639936bfa810bbe5d5.

7. The average number of respondents per country is 1,002, average age of participants was 49.0 years, 53% of respondents were female, and 38.6% reported attaining a bachelor’s degree or higher. For the wording of the survey questions, see the Supporting Information.

8. Concerning “denazification” narratives: https://www.state.gov/disarming-disinformation/to-vilify-ukraine-the-kremlin-resorts-to-antisemitism/; concerning the biolab conspiracy: https://www.state.gov/the-kremlins-allegations-of-chemical-and-biological-weapons-laboratories-in-ukraine/

9. The average rating of this claim ranges from 3.2 in Sweden to 4.9 in Greece (closely trailed by Serbia, Brazil, and Romania).

10. Specifically, we control for the following beliefs: 1) the Russian invasion was legitimate; 2) Russia should not be sanctioned for their military operation against Ukraine; 3) Russia uses disproportionate violence against Ukraine; 4) the global community should do more to help Ukraine to defend itself. Responses were rescaled to range from zero to one.

11.
https://www.newyorker.com/culture/infinite-scroll/watching-the-worlds-first-tiktok-war

12.
https://mitsloan.mit.edu/ideas-made-to-matter/russia-ukraine-war-social-media-stokes-ingenuity-disinformation

13. These calculations are based on the relative difference of coefficients on Facebook use in the first vs. sixth specification in Table 1.

14. Participants were reminded at different points in the survey and in different ways that there was no right or wrong answer and that we were interested in their opinions or best guesses.

15. Responses from plausibly inattentive respondents were not used in the analysis (see the SI for details), an imperfect solution which introduces certain tradeoffs (e.g., overall data quality is likely increased while representativeness is potentially reduced).

16. Reliance on self-reported data for media consumption means that media diets are also measured with a degree of noise.

## Data Sharing

Data, documentation, and code are made available via the Open Science Framework (OSF) at this repository: http://osf.io/szpj4/.

## Open scholarship

This article has earned the Center for Open Science badges for Open Data and Open Materials through Open Practices Disclosure. The data and materials are openly accessible at https://osf.io/szpj4/.
