# Supplementary material for: Justifying an Invasion: When Is Disinformation Successful?
Source: Polit Commun. 2024 May 22;41(6):965–86. doi: 10.1080/10584609.2024.2352483 (PMC11573309; doi:10.1080/10584609.2024.2352483)
Supplement: Supplemental Material [file UPCP_A_2352483_SM7448.pdf]

Supporting Information

Question wording

Outcome variables

The outcomes variables are the assessments of two factual claims, which were elicited as follows:

You may have come across many different statements, headlines and updates about the war in Ukraine. Below you see statements that have been circulating about the war. Could you rate the truthfulness of these statements? Please give us your best guess. A guess is just as good as the "right" answer.

- Ukraine's government is antisemitic and controlled by neo-Nazis.
- The U.S. is funding biological weapons research in Ukraine.

The ratings for the claim range from 1 to 5: (1) Very certain it's false, (2) Somewhat certain it's false, (3) Uncertain whether it's true or false, (4) Somewhat certain it's true, (5) Very certain it's true. The responses to the two items are averaged.

Independent variables

Conspiracy thinking is an additive scale (rescaled to range from 0 to 1) consisting of responses (on a 7-point scale, were 7 indicates strong agreement) to the following prompts: 1) Much of our lives are being controlled by plots hatched in secret places. 2) Even though we live in a democracy, a few people will always run things anyway. 3) The people who really "run" the country are not known to the voters. 4) Big events like wars and the outcomes of elections are controlled by small groups of people who are working in secret against the rest of us.

Political cynicism is an additive scale (rescaled to range from 0 to 1) consisting of responses to the following statements (1 = completely disagree; 7 = completely agree). 1) People are very frequently manipulated by politicians. 2) Politicians are only interested in getting and maintaining power. 3) Politicians pretend to care more about people than they really do. 4) Our political leaders are prepared to lie to us whenever it suits their purposes. 5) If a politician sticks to their ideals and principles, they are unlikely to reach the top of their profession. 6) No one can hope to stay honest once they enter politics. 7) Almost all politicians will sell out their ideals or break their promises if it will increase their power. 8) All politicians are bad – some are just worse than others.

Media use was elicited with the following prompt: *In your everyday life, how often do you get information about political news and societal issues from the following sources? (1 never - 7 very often) (8 = I don't use this source at all). Note that it doesn't matter through which device*

you get to the media or platform (smartphone, laptop, tablet, etc.). In the paper use responses to these items: Television; Newspapers and magazines (including news websites); social media (e.g., Facebook, Twitter, YouTube etc.).

Platforms: Respondents who indicated that they used social media at least occasionally to access news were also asked: When on social media, how often do you get information about political news and societal issues from the following source (1 = never; 7 = very often). The menu of platforms included Facebook, Twitter, Instagram, YouTube, and TikTok.

When included as predictors, all media consumption and platform variables were recoded to range from zero to one.

Political predictors:

- Political orientation (0 to 11; left-right ideological self-identification)
- Ideological strength: folded ideology, i.e. the distance from the center
- Political interest (1-7 scale ranging from "not at all interested" to "very interested" in politics)

The "pro-Russia sentiment" taps into approval of Russia's military action in early 2022, and its components are responses to the following questions: 1) Russia's military operation in Ukraine is legitimate, 2) Russia should not be sanctioned for their military operation against Ukraine, 3) Russia uses disproportionate violence against Ukraine (reverse-coded), 4) the global community should do more to help Ukraine to defend itself (reverse-coded).

Quality/attentiveness checks

Kantar removed 2,976 respondents who finished the survey in less than five minutes. This cut-off point was chosen because it represented less than 40% of the median completion time, and this is the typical approach that the survey company would typically follow. The pollster also removed 234 respondents who had completion times of more than 2 hours. (This was upon our request and had to do with analyses for a separate paper on the effects of reading fact-checks.) Accordingly, 14.4% ((2976 + 234) / (19037 + 2976 + 234)) of respondents were dropped due to potential inattentiveness. The quotas of min. 1,000 respondents per country were met in all countries.

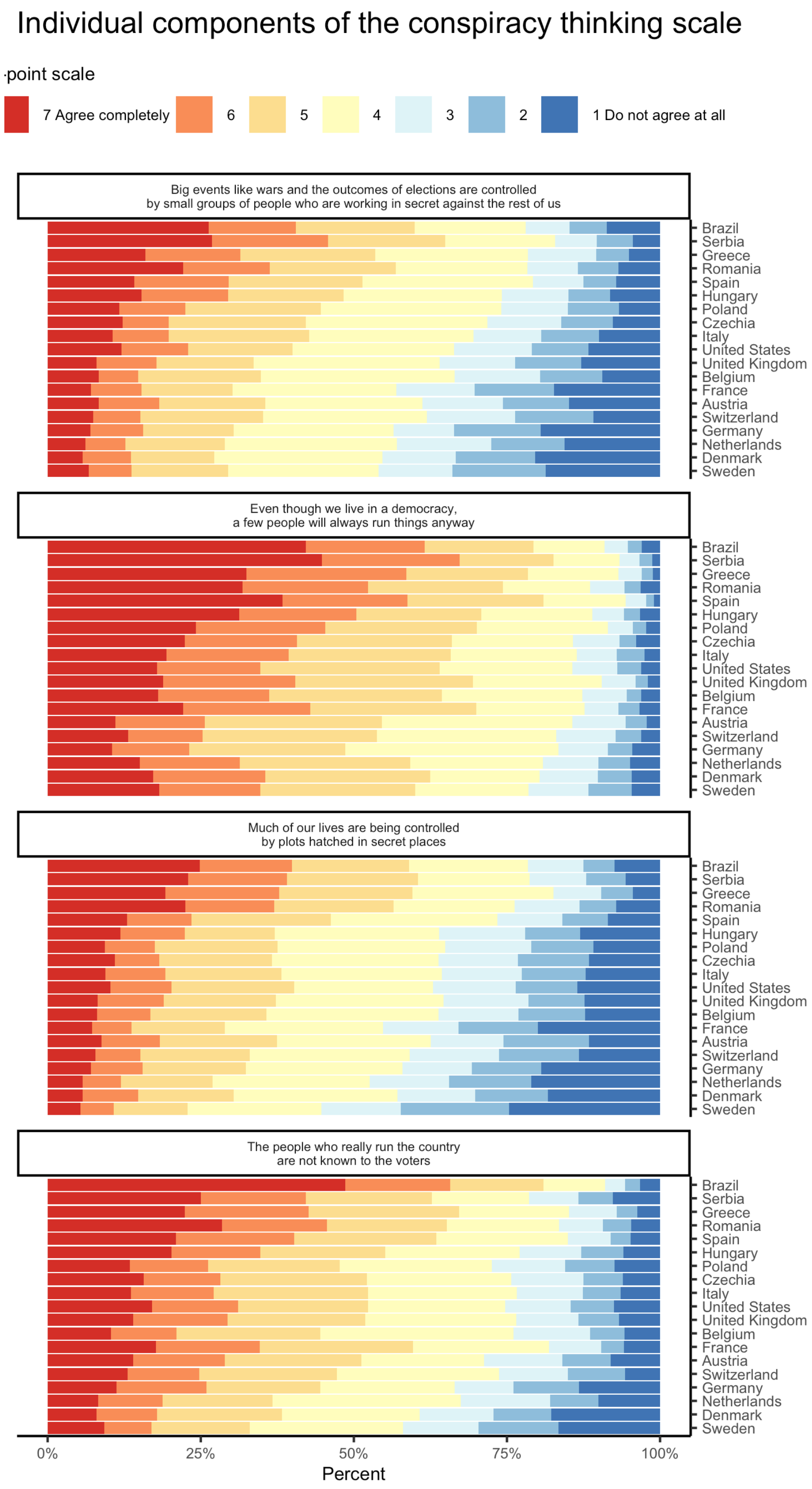

Figure S.1: Components of conspiracy thinking by country

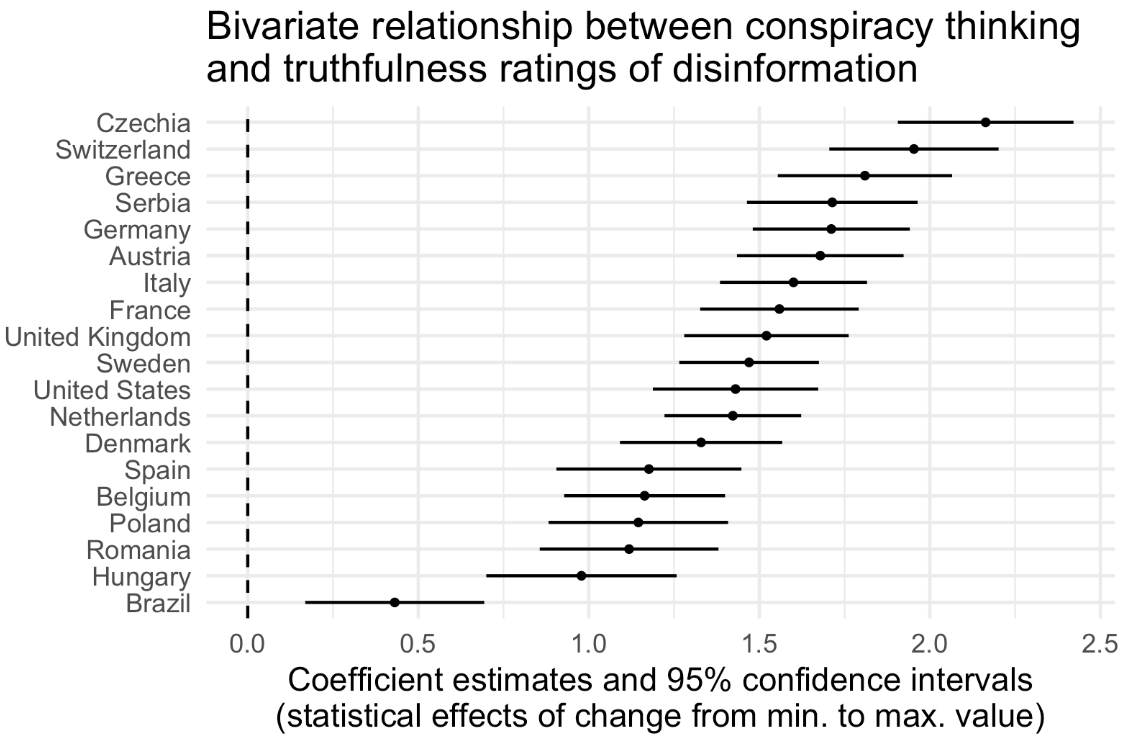

Figure S.2: Bivariate relationship between conspiracy thinking and average belief in disinformation (the LHS variable ranges from 1 to 5 where 1 indicates certainty that propaganda claims are false and 5 indicates certainty that they are true).

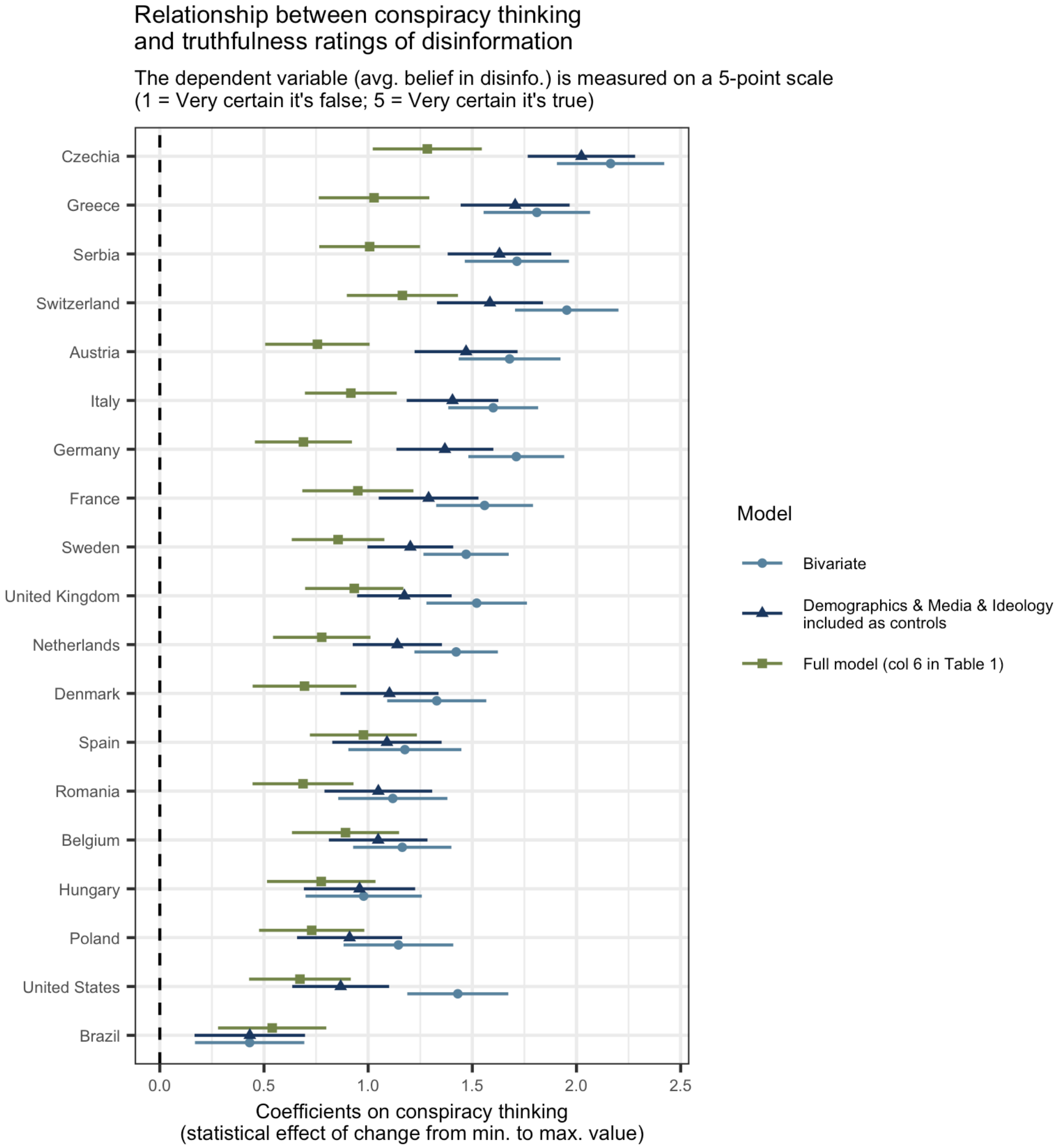

Figure S.3: Relationship between conspiracy thinking and average belief in disinformation: 3 specifications, corresponding to models 1, 5 and 6 in Table 1. Model condition on media consumption (incl. 5 platforms), age, gender, education, and cynicism.

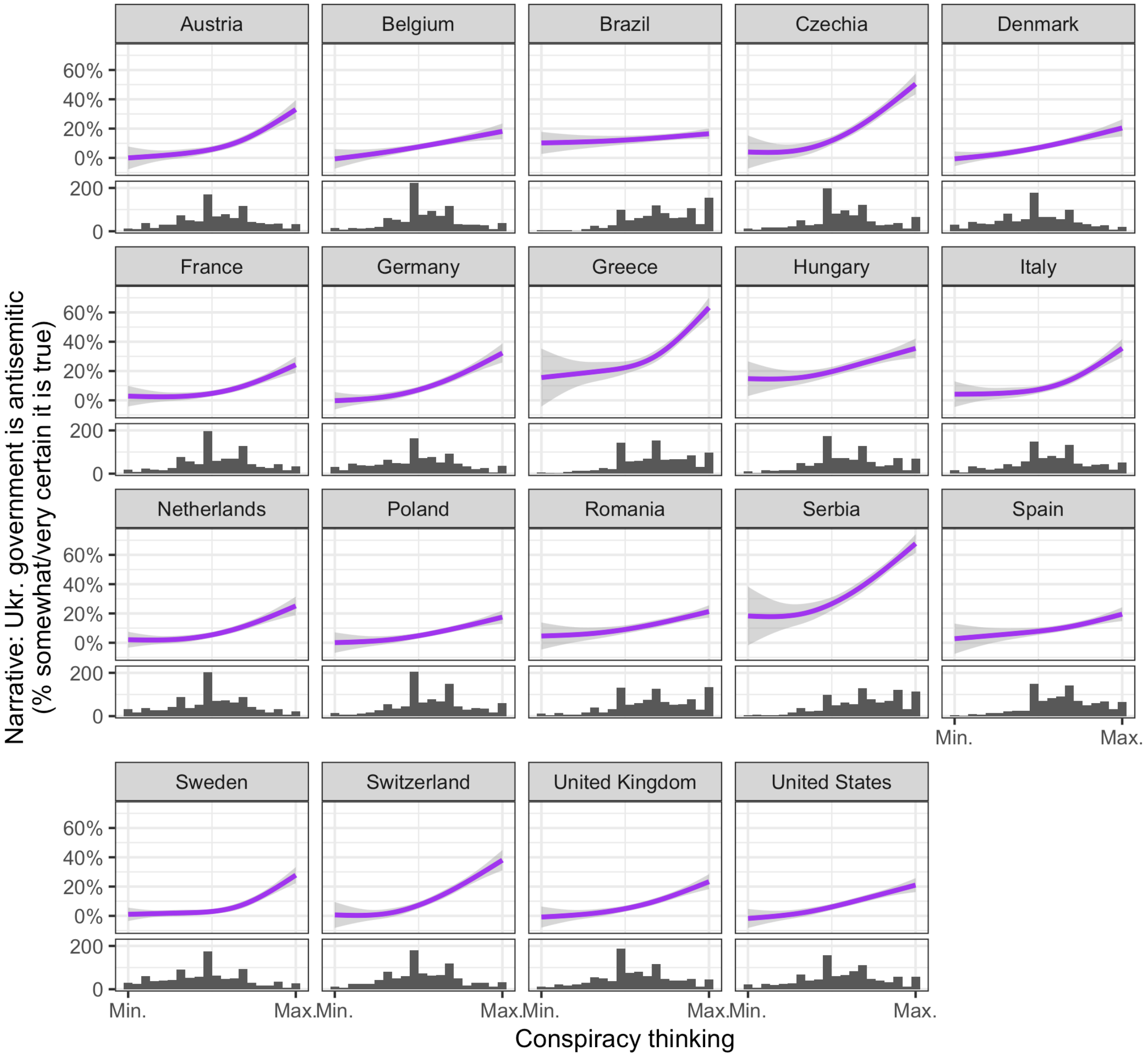

Figure S.4: Bivariate relationship between the belief that Ukraine's government is antisemitic and controlled by neo-Nazis and the predisposition to engage in conspiracy thinking.

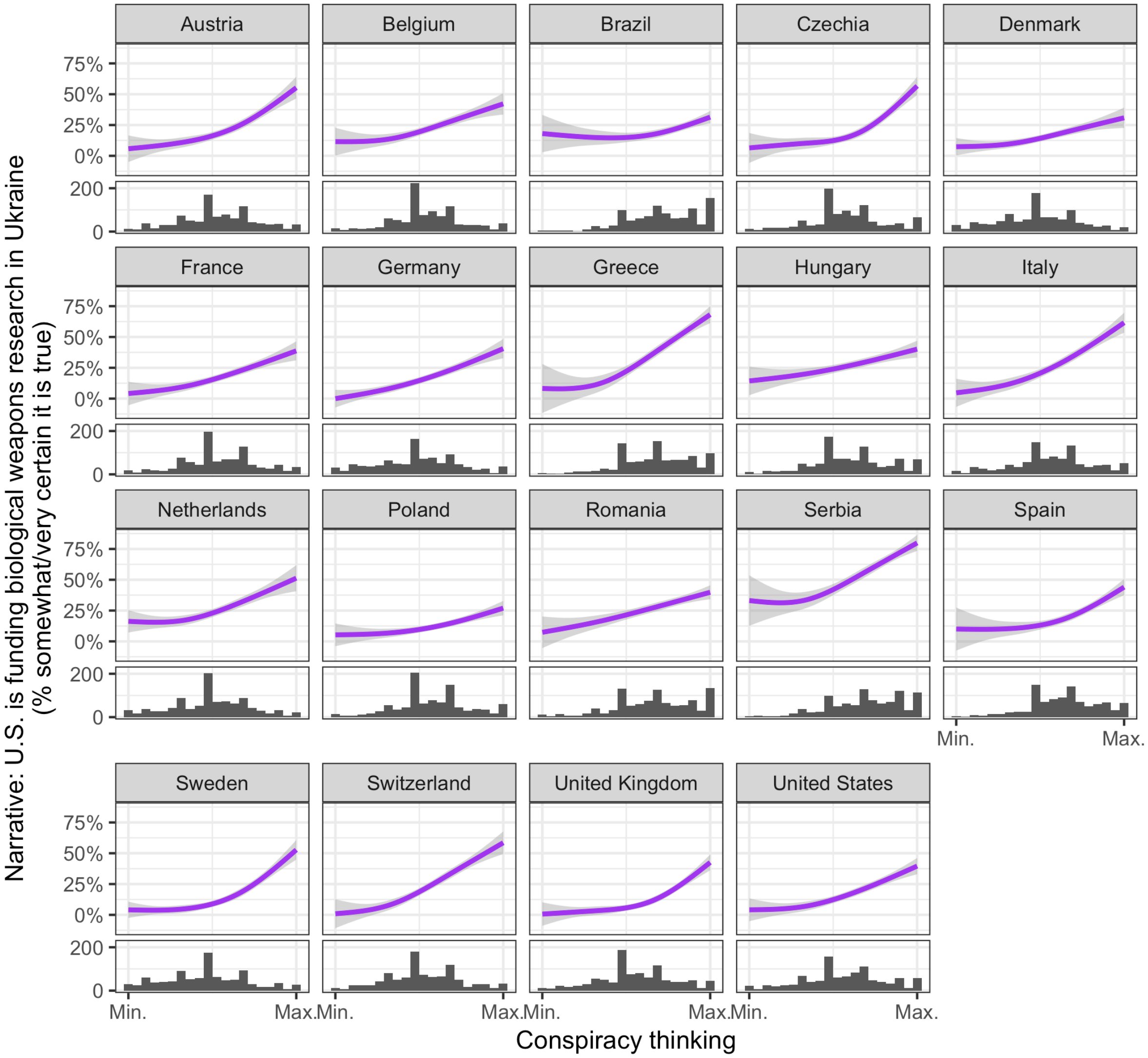

Figure S.5: Bivariate relationship between the belief that the U.S. is funding biological weapons research in Ukraine and the predisposition to engage in conspiracy thinking.

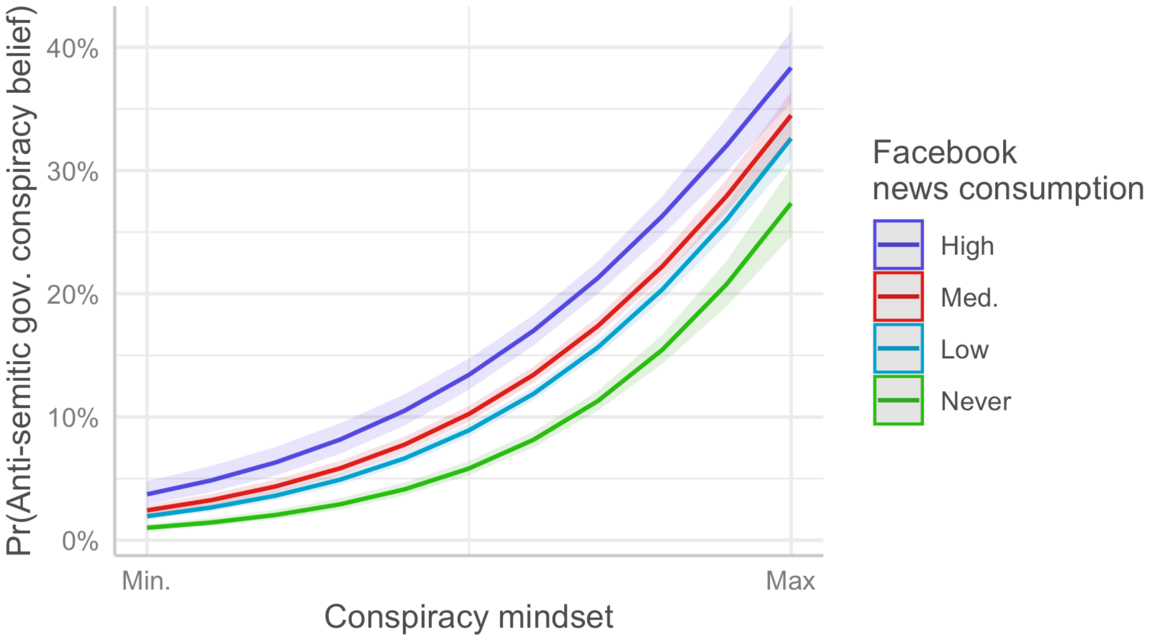

Figure S.6: Belief that the Ukrainian government is anti-Semitic as a function of conspiracy thinking, interacted with getting news via Facebook. Standard control variables (gender, age, education, political interest, and political orientation) are included in the model.

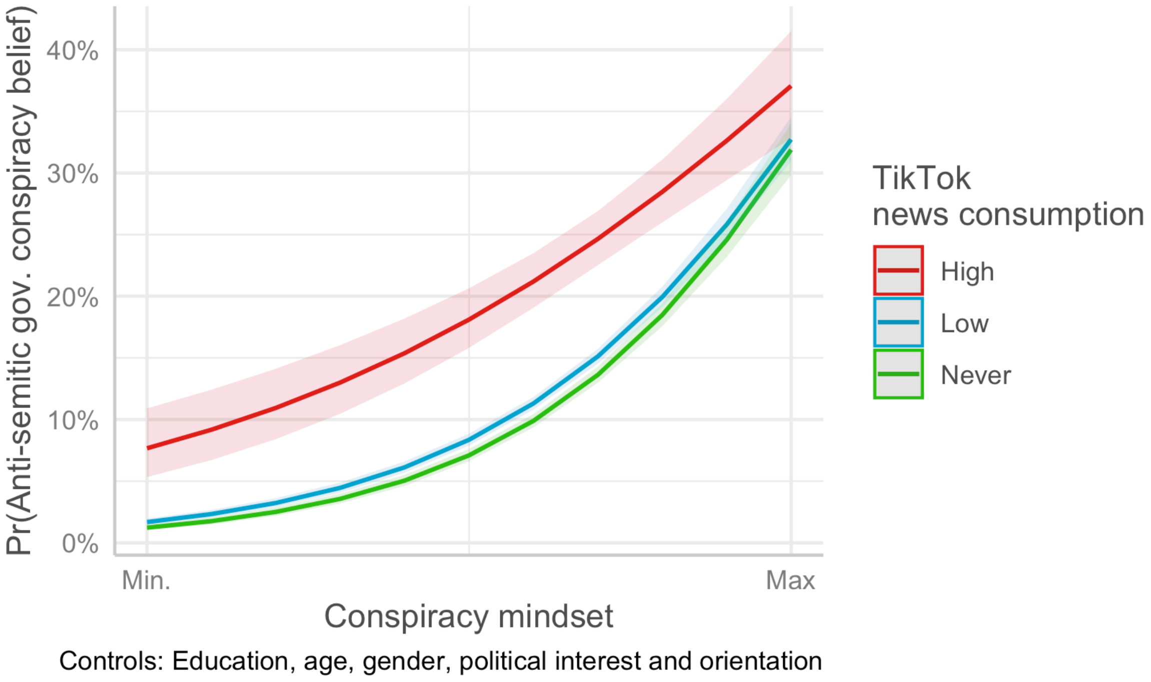

Figure S.7: Belief that the Ukrainian government is anti-Semitic as a function of conspiracy thinking, interacted with getting news via TikTok. Standard control variables (gender, age, education, political interest, and political orientation) are included in the model.

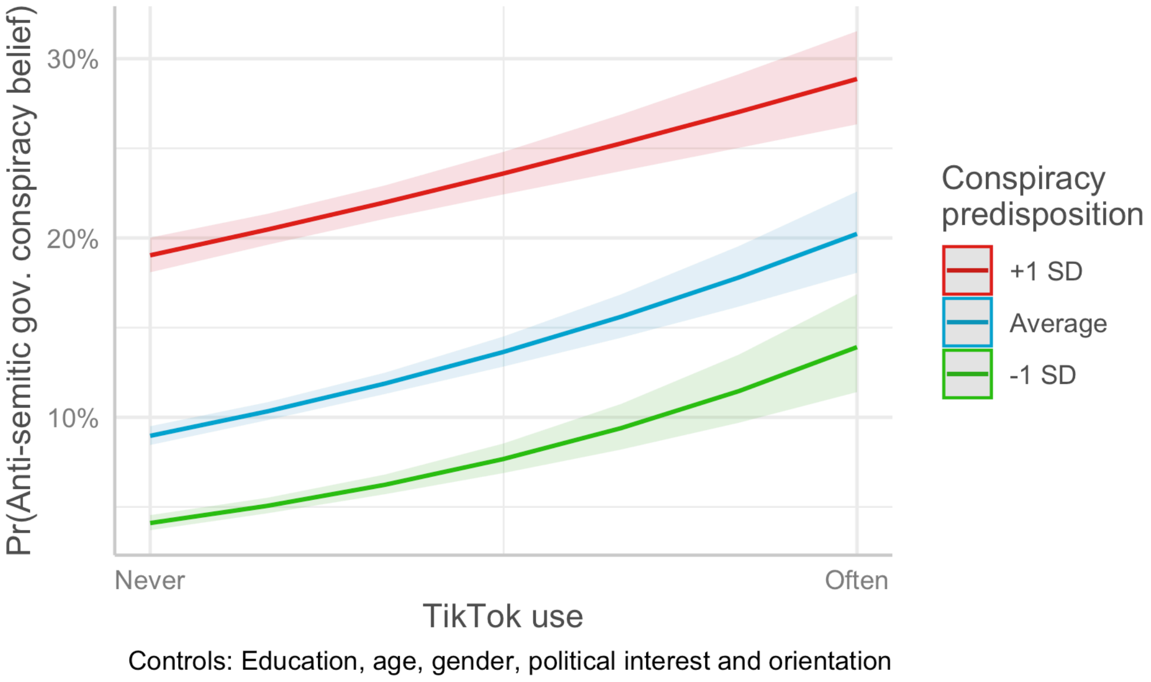

Figure S.8: Belief that the Ukrainian government is anti-Semitic as a function of TikTok use (for news) interacted with conspiracy thinking. Standard control variables (gender, age, education, political interest, and political orientation) are included in the model.

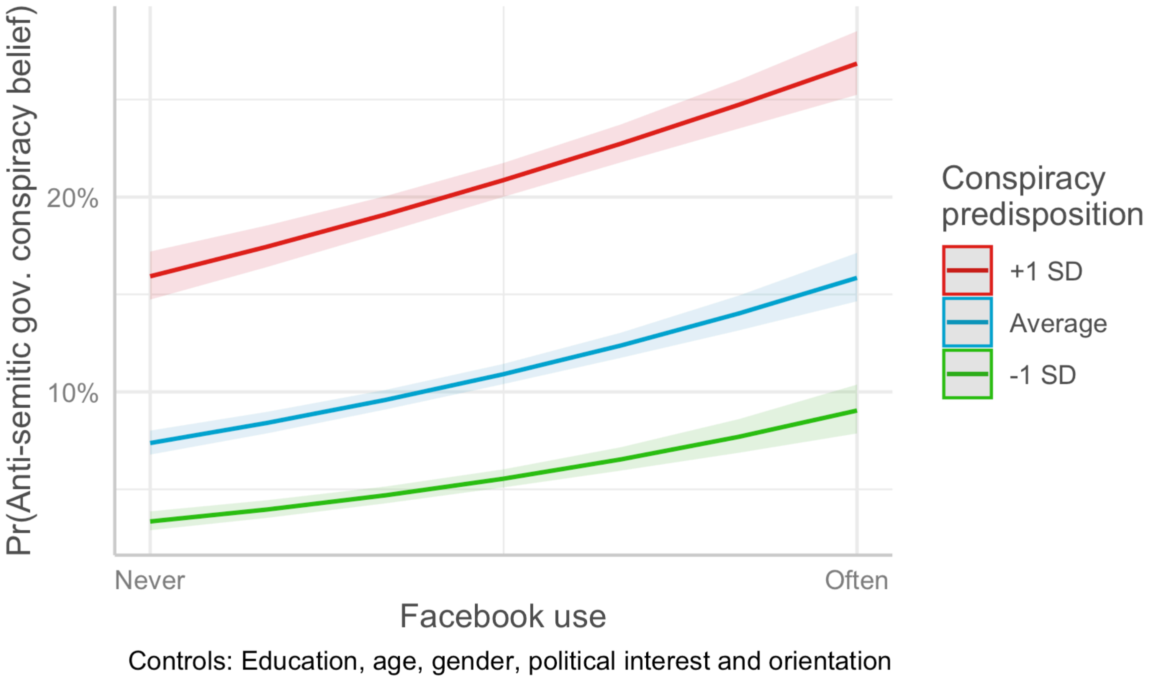

Figure S.9: Belief that the Ukrainian government is anti-semitic as a function of Facebook use (for news) interacted with conspiracy thinking. Standard control variables (gender, age, education, political interest, and political orientation) are included in the model.

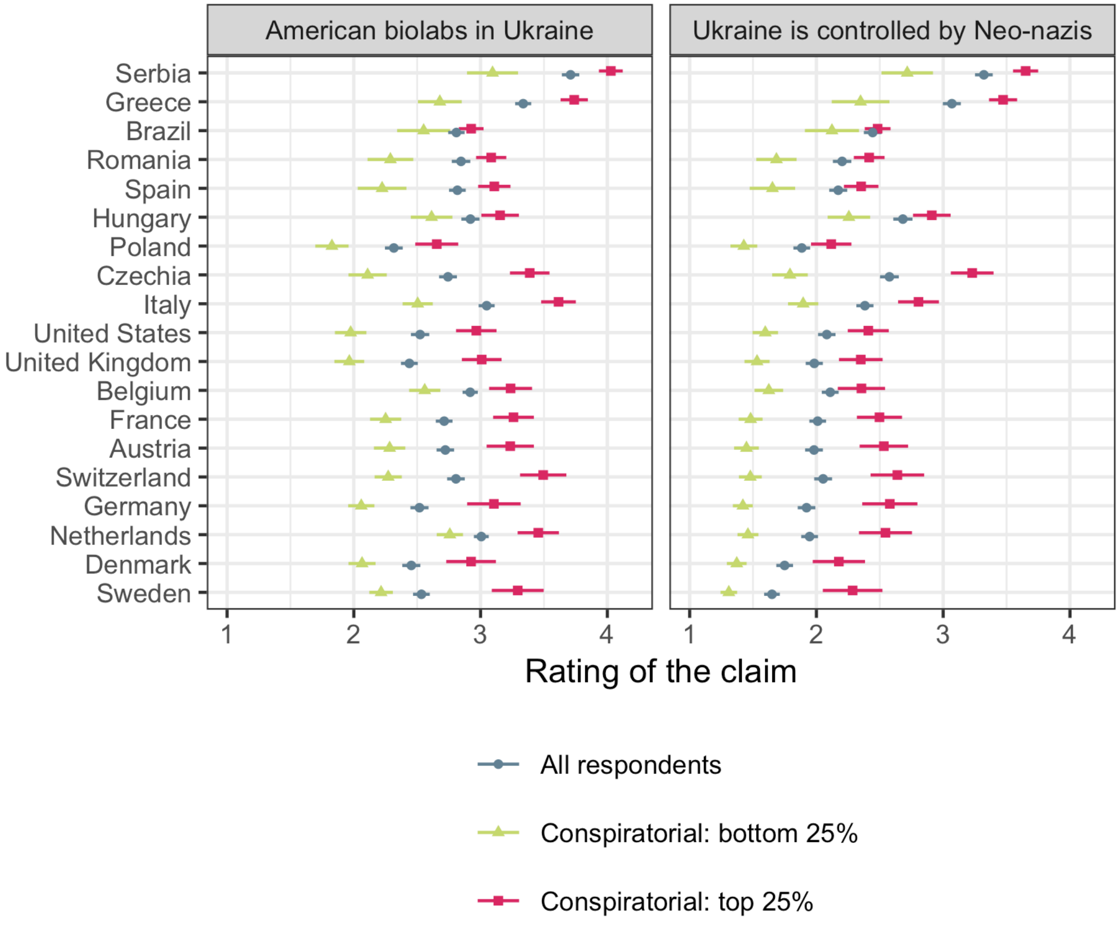

Figure S.10: Ratings of the two narratives, broken down by the level of conspiracy thinking.

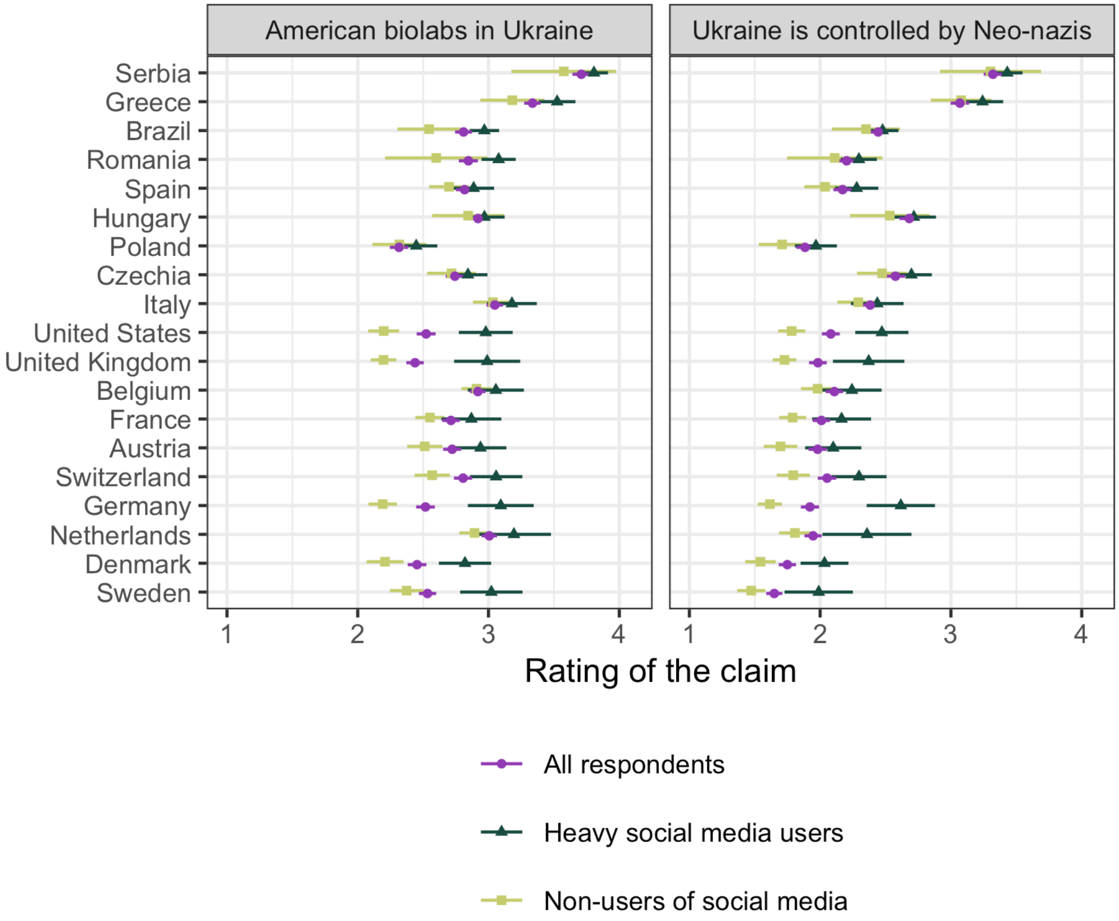

Figure S.11: Ratings of the two narratives, broken down by the level of news consumption via social media.

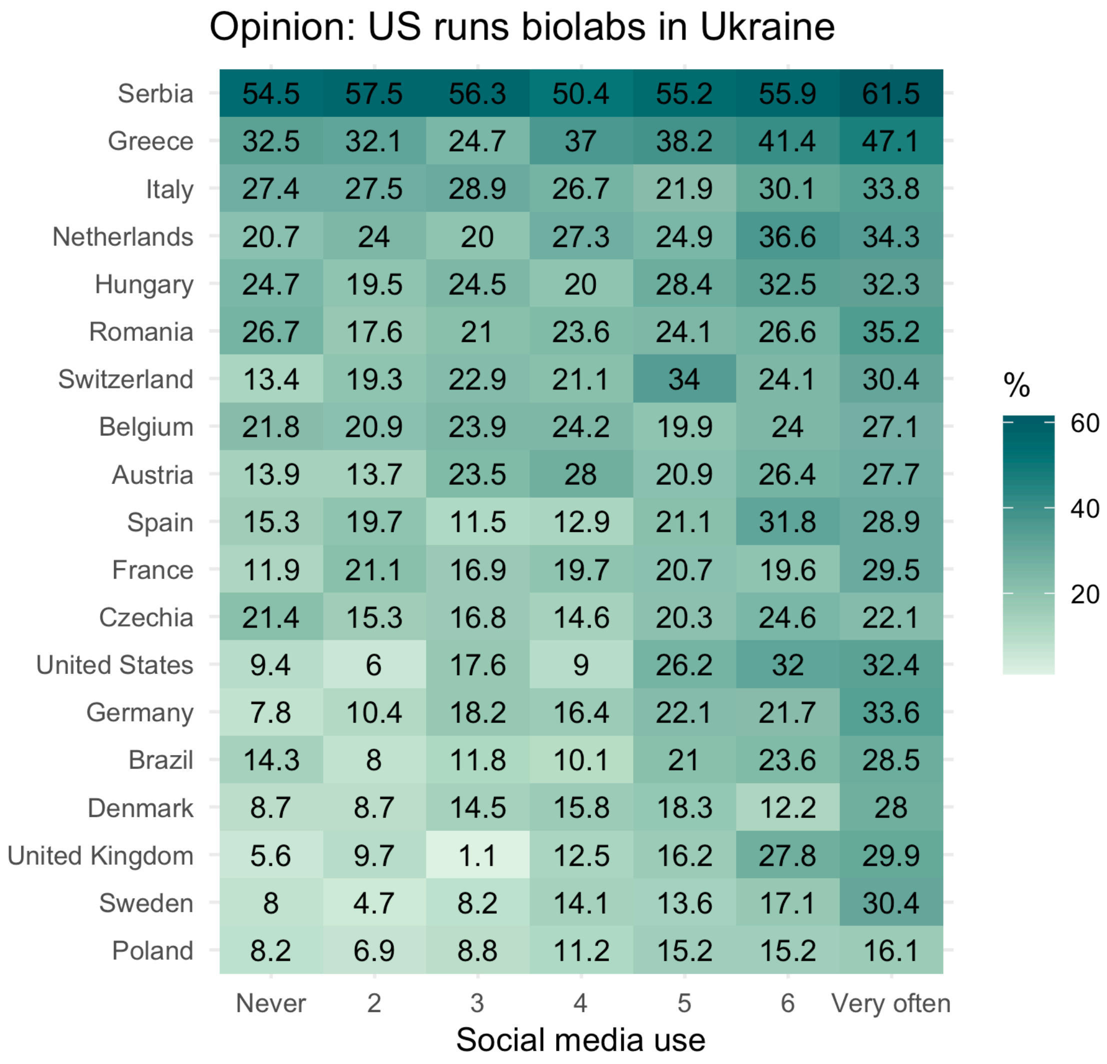

Figure S.12: Average endorsement of the biolab conspiracy theory by the level news consumption via social media

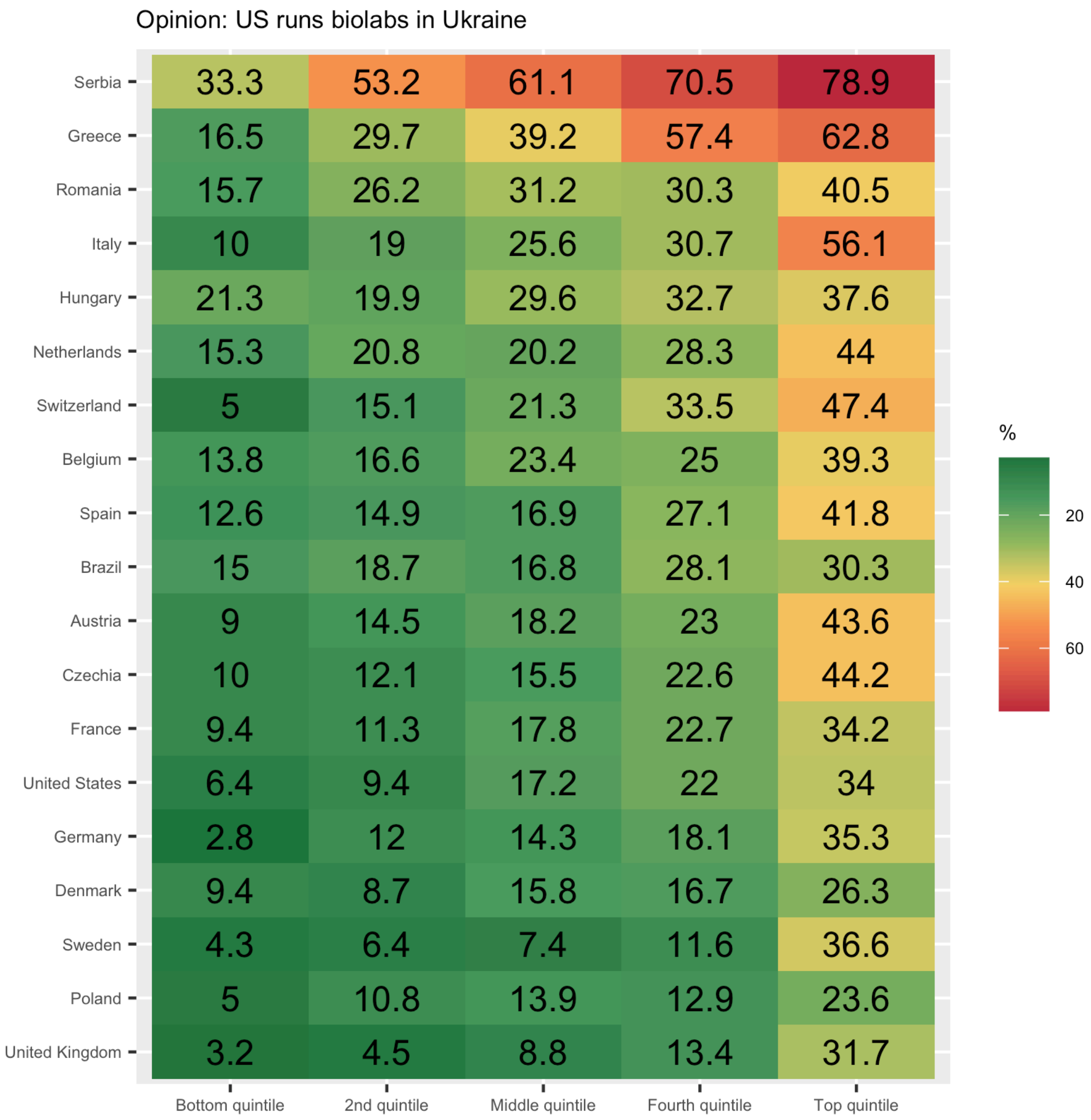

Figure S.13: Average endorsement of the biolab conspiracy theory by (within-country) quintile of conspiracy thinking

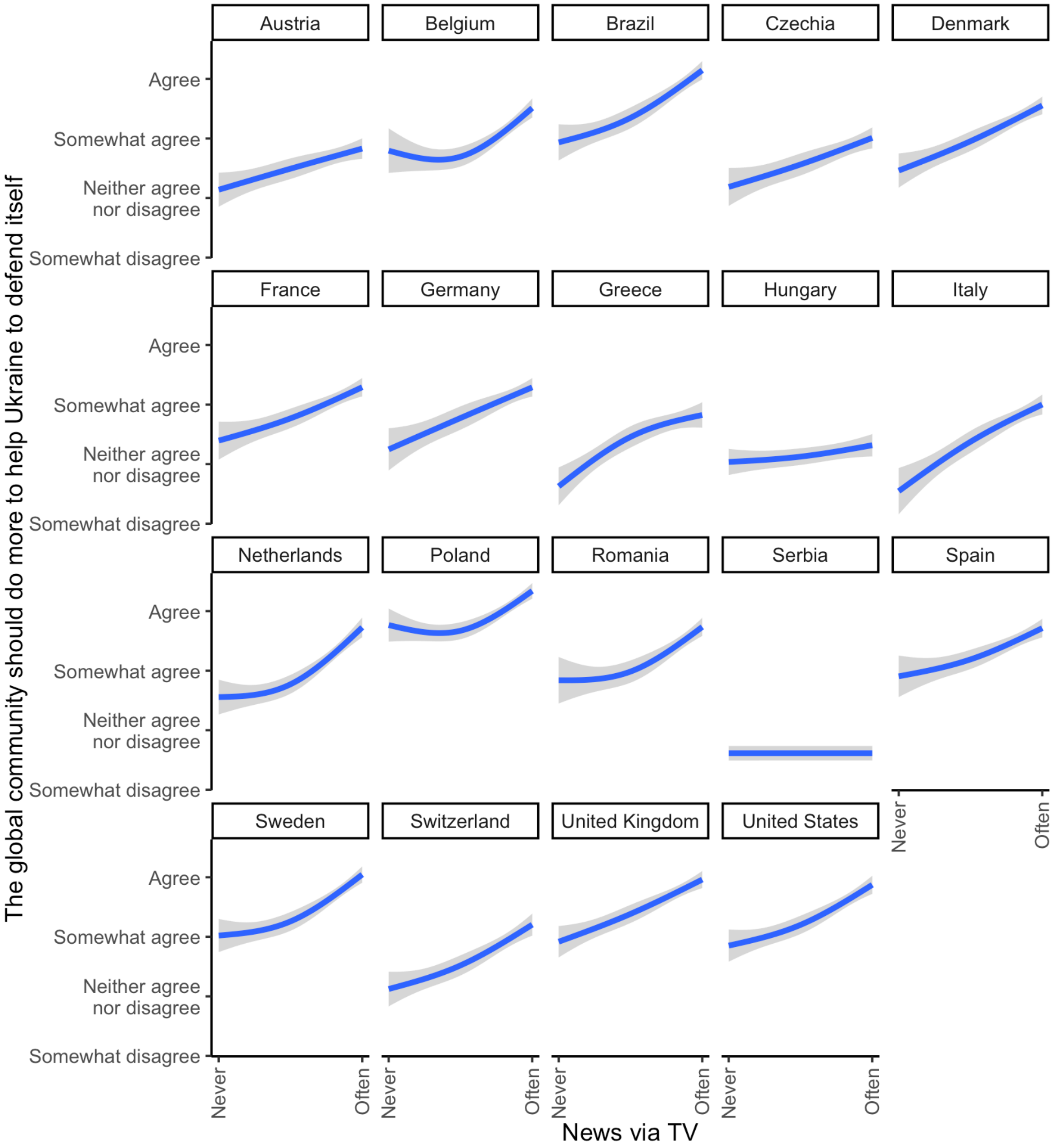

Figure S.14: A bivariate relationship between getting news via TV and believing that Ukraine should receive more support from the global community.

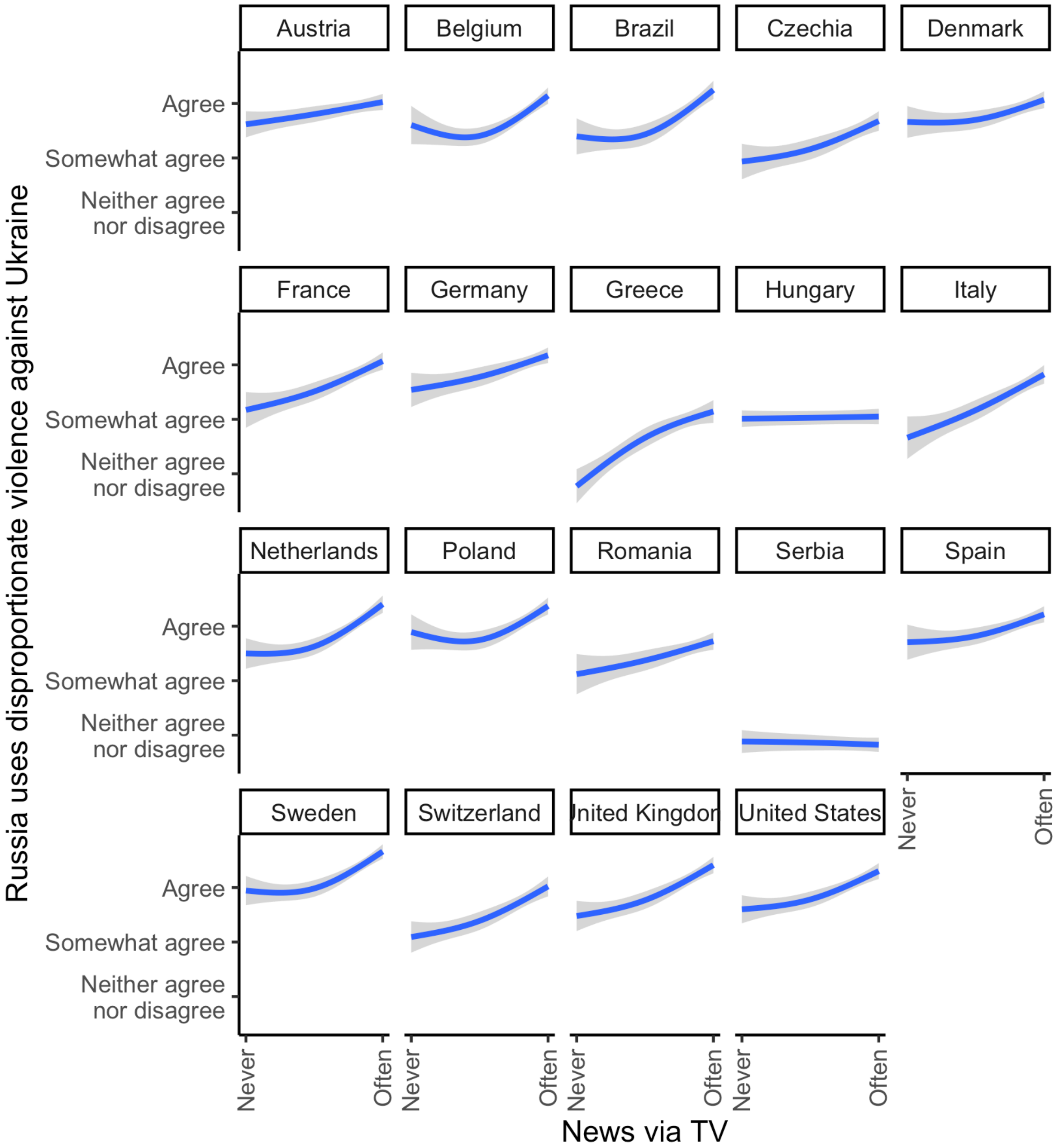

Figure S.15: A bivariate relationship between getting news via TV and believing that Russia uses disproportionate violence against Ukraine.

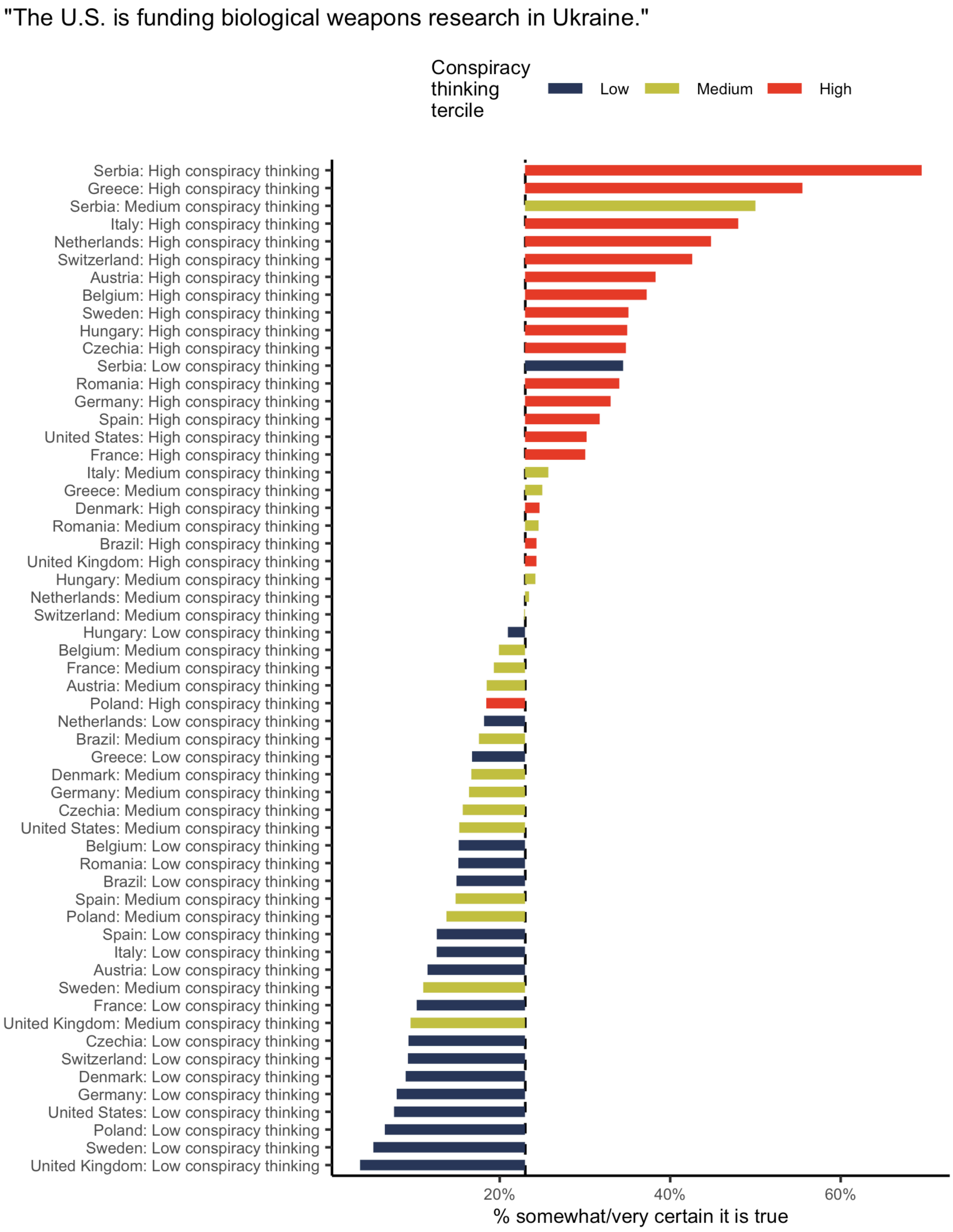

Figure S.16: Deviation from the global opinion that the U.S. is funding biological weapons research in Ukraine (average belief in the pooled sample is 23%). Respondents are broken down into thirds on the basis of their conspiracy thinking predisposition.

Country	Women (%) (*survey participants*)	65+ years old (%) (*survey participants*)	High Education (%) (*survey participants*)	Women (%) (*official statistics*)	65+ years old (%) (*official statistics*)	High Education (%) (*official statistics*)
Austria[a]	53	17	28	52	19	32
Belgium[a]	52	25	43	51	19	40
Brazil[b]	54	15	47	51	10	21
Czech Republic[a]	52	9	27	51	20	23
Denmark[a]	48	29	36	51	20	35
France[a]	54	29	36	51	21	36
Germany[a]	51	28	26	51	22	27
Greece[a]	52	2	52	50	23	30
Hungary[a]	54	9	35	52	20	25
Italy[a]	51	26	33	52	24	18
Netherlands[a]	57	27	31	51	20	38
Poland[a]	53	18	44	52	19	29
Romania[a]	49	4	62	51	19	16
Serbia[b]	51	9	44	51	29	22
Spain[a]	51	19	40	51	20	36
Sweden[a]	59	31	36	51	20	40
Switzerland[a]	52	17	40	51	19	39
United Kingdom[b]	55	30	38	51	19	41
United States[b]	52	39	48	51	17	38

a: The official statistics on the percent of women, +65 years old and high education (tertiary education) were derived from Eurostat. As high education (tertiary education) is considered any level of education pursued beyond high school.

b: The official statistics on the percent of women were taken from the World Bank. Age data is from UN Nations Population Division. For education, we used census data for United States (US census bureau), data from OECD for Brazil and OECD data for the United Kingdom which calculates high education (those who attained tertiary education, i.e., bachelor degree or above) among 25-64 year-olds. The statistics for Serbia were taken from Eursostat.

Table SI.1: Sociodemographic characteristics of participants of the survey data in comparison to populations' sociodemographic characteristics based on official statistics.

Table S.2: Relationships between observables and truthfulness ratings of disinformation (index ranging from 1 to 5). All models include country fixed effects.

	(1)	(2)	(3)
Univ. graduate	-0.170** (0.014)	-0.115** (0.014)	-0.081** (0.014)
Female respondent	0.168** (0.014)	0.159** (0.013)	0.149** (0.013)
Age: 18-24	0.140** (0.029)	0.178** (0.027)	0.004 (0.029)
25-34	0.170** (0.024)	0.158** (0.022)	0.044 (0.023)
35-44	0.104** (0.022)	0.092** (0.021)	0.031 (0.021)
55-64	-0.071** (0.022)	-0.068** (0.021)	-0.014 (0.020)
65-74	-0.182** (0.024)	-0.180** (0.022)	-0.074** (0.022)
75 or older	-0.245** (0.029)	-0.246** (0.027)	-0.125** (0.027)
Conspiracy thinking		1.422** (0.031)	1.273** (0.031)
Platform: Facebook			0.119** (0.025)
Platform: Youtube			0.170** (0.028)
Platform: TikTok			0.277** (0.032)
Platform: Instagram			0.015 (0.030)
Platform: Twitter			0.036 (0.027)
News: TV			-0.238** (0.024)
News: Newspapers			-0.115** (0.022)
Political Interest			-0.230** (0.026)
Political Orientation			0.224** (0.027)
Ideological strength			0.008 (0.021)
Num.Obs.	19037	19037	19037
R2	0.156	0.255	0.284
RMSE	0.93	0.87	0.86

Standard errors in parentheses. Stars denote $p < .05$, and $p < .01$ respectively.
